# Multiplexed Spliced-Leader Sequencing: A high-throughput, selective method for RNA-seq in *Trypanosomatids*

**DOI:** 10.1038/s41598-017-03987-0

**Published:** 2017-06-16

**Authors:** Bart Cuypers, Malgorzata A. Domagalska, Pieter Meysman, Géraldine de Muylder, Manu Vanaerschot, Hideo Imamura, Franck Dumetz, Thomas Wolf Verdonckt, Peter J. Myler, Gowthaman Ramasamy, Kris Laukens, Jean-Claude Dujardin

**Affiliations:** 1 Molecular Parasitology Unit, Department of Biomedical Sciences, Institute of Tropical Medicine, Antwerp, Belgium; 2 Advanced Database Research and Modeling group (ADReM), Department of Mathematics and Computer Science, University of Antwerp, Antwerp, Belgium; 3 Center for Infectious Disease Research, Seattle, Washington, United States of America; 4 Department of Global Health and Department of Biomedical Informatics & Medical Education, University of Washington, Seattle, Washington, United States of America; 5 Department of Biomedical Sciences, University of Antwerp, Antwerp, Belgium; 6 Fidock Lab, Department of Microbiology, College of Physicians and Surgeons, Columbia University, New York, USA

## Abstract

High throughput sequencing techniques are poorly adapted for *in vivo* studies of parasites, which require prior *in vitro* culturing and purification. Trypanosomatids, a group of kinetoplastid protozoans, possess a distinctive feature in their transcriptional mechanism whereby a specific Spliced Leader (SL) sequence is added to the 5′end of each mRNA by *trans-splicing*. This allows to discriminate Trypansomatid RNA from mammalian RNA and forms the basis of our new multiplexed protocol for high-throughput, selective RNA-sequencing called SL-seq. We provided a proof-of-concept of SL-seq in *Leishmania donovani*, the main causative agent of visceral leishmaniasis in humans, and successfully applied the method to sequence *Leishmania* mRNA directly from infected macrophages and from highly diluted mixes with human RNA. mRNA profiles obtained with SL-seq corresponded largely to those obtained from conventional poly-A tail purification methods, indicating both enumerate the same mRNA pool. However, SL-seq offers additional advantages, including lower sequencing depth requirements, fast and simple library prep and high resolution splice site detection. SL-seq is therefore ideal for fast and massive parallel sequencing of parasite transcriptomes directly from host tissues. Since SLs are also present in Nematodes, Cnidaria and primitive chordates, this method could also have high potential for transcriptomics studies in other organisms.

## Introduction

Trypanosomatidae are a family of protozoan organisms that comprise a variety of human and animal pathogenic species including human/animal African trypanosomiasis or sleeping sickness (*Trypanosoma brucei*), Chagas disease (*Trypanosoma cruzi*) and surra (*Trypanosoma evansi*). An important genus within the Trypanosomatid family is *Leishmania*, responsible for a wide array of diseases in humans, ranging from self-healing cutaneous lesions to lethal visceral leishmaniasis. Globally, the incidence of this disease is between 0.9–1.6 million cases each year^1^. Transmission occurs via the sandfly vector in which two main parasite life stages can be distinguished: procyclic promastigotes, a replicative stage occurring in the midgut, and infectious metacyclic promastigotes that reside in the proboscis. Humans can be infected when metacyclics are injected into the bloodstream during a sand fly bloodmeal. These metacyclics then infect mononuclear phagocytes and convert to the amastigote life stage that is able to survive within the phagolysosome by subverting the host cell and modulating the immune system. Therapeutic options for leishmaniasis are limited, with only 4 drugs available and drug resistance emerging rapidly^2^. Transcriptome studies of these intracellular parasites are therefore important to improve the understanding of host-pathogen interactions, characterize drug-resistance and potentially find molecular targets for new therapeutic agents. However, *in vivo*, host cell RNA is much more abundantly present than parasite RNA. Parasite isolation/purification is therefore required prior to sequencing.

Transcription in Trypanosomatidae and other Kinetoplastida differs from most other Eukaryotes^3^. Genes are not under individual regulation of RNA polymerase II promotors, but constitutively transcribed in large polycistronic units^4^. Transcription is followed by trans-splicing and polyadenylation to split these long transcripts into individual mature mRNAs per gene. During trans-splicing the first 39 nucleotides from a spliced leader (SL) RNA or mini exon are post-transcriptionally spliced onto the 5′ end of each mRNA^5^. In contrast to conventional splicing (cis-splicing) mechanisms, the spliced leader originates from a different genomic location, which contains tandem repeats of mini exon genes. Polycistronic units are transcribed constitutively, thus mRNA levels are presumed to be regulated post-transcriptionally by mRNA stability and decay. However, striking mRNA changes have been observed in transcriptomics studies comparing different *Leishmania* life stages. For example more than 30% of the genes were differentially expressed between procyclic and metacyclic promastigotes^6^. This suggests a strong, yet currently unknown stage-specific regulation mechanism. Interestingly, Nilsson *et al*. (2010) hypothesized that trans-splicing itself could impact mRNA stability^7^. Multiple SL-addition sites were observed for single genes, some of them being differentially used between different life stages. Since the SL-addition site determines the 5′-UTR length, altering these frequencies could impact stage-specific mRNA turnover.

The presence of a SL can be used to specifically amplify Trypanosomatid mRNA and therefore offers interesting opportunities for RNA-seq strategies. An elegant method called ‘Spliced-Leader Trapping’^7^ and a set of derived protocols^8–10^ have been developed for this purpose. The key feature of SL Trapping is that it uses a combination of a random and a SL primer during cDNA synthesis to select specifically for the mRNA from these parasites for subsequent sequencing library preparation. This method has several major advantages compared to conventional RNA-seq: (1) It has the potential to sequence parasite mRNA directly from infected tissue, without prior purification. However, to our knowledge, this had not been validated yet. (2) Every sequenced Trypanosomatid read corresponds to the splice location of a SL. As the SL-addition site is presumed to play a role in regulation, its in depth study could offer major advantages to ‘omics and systems biology studies. Additionally, this high resolution mapping of the SLs offers a major advantage towards annotation of the 5′-UTRs, which is problematic in *Trypanosomatidae* and reflected in the poor or even absent annotations for UTRs in Trypanosomatid reference genomes^11^. Indeed, the polycistronic transcription and the presence of multiple SL splicing sites for a single gene make the annotation of 5′ UTRs very difficult with conventional means^12^. (3) The method is relatively inexpensive because it requires no commercial library prep kits. Although this method is very promising, it has not been widely used for a number of reasons. Firstly, SL trapping was developed for the Illumina Genome Analyzer, but is incompatible with recent Illumina sequencers like HiSeq and NextSeq, which are able to generate much more sequencing reads per lane. Secondly, sequencing data obtained with SL Trapping slightly differs from conventional RNA-seq data, since sequencing reads map often just upstream of the gene (5′ UTR), where the spliced leader is added. This requires a bioinformatics pipeline that is adapted and benchmarked for this type of data. Standard RNA-seq data analysis workflows only consider reads that map within the annotated regions, which for most Kinetoplastida are limited to the coding sequences and do not extend to the 5′ UTR. Finally, SL trapping has, to the best of our knowledge, never been technically or functionally compared against conventional RNA-seq.

We present a new protocol called SL-seq that builds on the original principles of Spliced Leader Trapping, addresses all the issues previously raised and validate it in *Leishmania donovani* as a proof of concept for *Trypanosomatidae*. Additionally, with SL-seq hundreds of samples can be uniquely indexed and pooled on the same sequencing lane, since it is adapted to the newest Illumina platforms. We benchmarked the method against conventional RNA-seq and demonstrated that it is highly specific and sensitive. In addition, we provide a bioinformatic pipeline for easy and reliable data-analysis. Using our SL-seq method, we were able to sequence *Leishmania* mRNA directly from infected THP-1 cells without prior purification and from samples diluted with high amounts of human RNA. This work could bring transcriptomics in parasitic Trypanosomatids to a new performance level, opening a novel avenue for in-depth understanding of their molecular regulation. Furthermore, this method also has high potential for *in vivo* studies of other symbionts with a SL-containing mRNA such as nematodes, trematodes, dinoflagellates and primitive chordates^13^.

## Material and Methods

### Parasites

The *Leishmania* strains used for this study are MHOM/NP/2003/BPK275/0cl18 (BPK275) and MHOM/NP/2003/BPK282/0cl4 (BPK282). Both are clinical *Leishmania donovani* strains, originating from Nepal and characterized at biological and (phylo-)genomic levels^14^. Unless stated otherwise, axenic promastigotes were cultured at 26 °C in HOMEM supplemented with 20% heat-inactivated fetal bovine serum (Biochrom). Exponentially growing promastigotes (LOG) were harvested on day 4, while stationary phase promastigotes were harvested on day 7.

### RNA extractions and sequencing of *L. donovani* LOG and STAT phase

Four biological growth replicates (BR) of both logarithmic (LOG) and stationary phase (STAT) BPK275 promastigotes were cultured according to the ‘Parasites’ section. LOG cultures were dominated by dividing, procyclic promastigotes, while STAT cultures contained a significant proportion of non-dividing parasites mimicking the metacyclic stage. The parasites were washed 3 times by centrifugation at 18,000 x *g* at 0 °C and resuspended in ice-cold PBS buffer. After a final centrifugation round and aspirating the PBS, RNA was extracted with the AllPrep DNA/RNA Mini Kit (QIAGEN) according to the manufacturer’s protocol. The RNA concentration was determined with the Qubit 2.0 Fluorometer and RNA integrity was verified with the Bioanalyzer 2100 (Agilent) using the Agilent RNA 6000 Nano Kit (Agilent). Sequencing libraries were prepared with both the stranded mRNA kit (Illumina) according to the manufacturer’s instructions and according to our new SL-seq protocol that is presented below in the section ‘Multiplexed Spliced Leader mRNA Sequencing’. These libraries were quantified with the Library Quantification Kit for Illumina systems (KAPA) and 2X100BP paired-end sequenced on an Illumina Hiseq 1500 (Center of Medical Genetics, Antwerp).

The same extraction protocol was repeated to extract and sequence RNA for another 3 replicates of BPK275 stationary promastigotes. For each replicate, dilutions with human RNA (Human Spleen Total RNA, ThermoFisher Scientific) were prepared at a ratio of 1:100 and 1:1000 (*Leishmania:*human) and 1X50BP sequenced on the HiSeq1500 platform.

### RNA sequencing of *Leishmania* intracellular amastigotes

THP-1 cells (ATCC TIB202) were treated with 0.1 μM phorbol myristate acetate (PMA, Sigma) at 37 °C for 48 h to achieve differentiation into adherent, non-dividing macrophages. Cells were washed and incubated with complete RPMI medium containing stationary phase *L. donovani* BPK275, BPK282 promastigotes (2 replicates each) at a macrophage:promastigote ratio of 1:10. Non-infected macrophages were included as control. After 4 h incubation at 37 °C, non-internalized promastigotes were removed by 3 successive washes with PBS and incubated for 24 h at 37 °C. Uninfected THP-1 cells were similarly treated in parallel. After 24 h incubation, RNA was extracted from uninfected and infected THP-1 cells with the RNAqueous micro kit (Ambion) following the manufacturer instructions. The RNA concentration was determined with the Qubit 2.0 Fluorometer and the RNA integrity was verified with the Bioanalyzer 2100 (Agilent) using the Agilent RNA 6000 Nano Kit (Agilent). 2 types of libraries were prepared in parallel: (i) library from total RNA of uninfected and infected THP-1 using the TruSeq stranded mRNA sample prep kit (Illumina) and (ii) *Leishmania* RNA enriched library using the SL-seq protocol. The Illumina library was paired-end sequenced (2 × 100BP) on an Illumina Hiseq 1500 and the SL-seq library single read sequenced (1X50BP) on the same machine. Both libraries were quantified with the KAPA Library Quantification kit for Illumina systems prior to sequencing.

### Multiplexed Spliced Leader mRNA Sequencing

**Figure 1:**
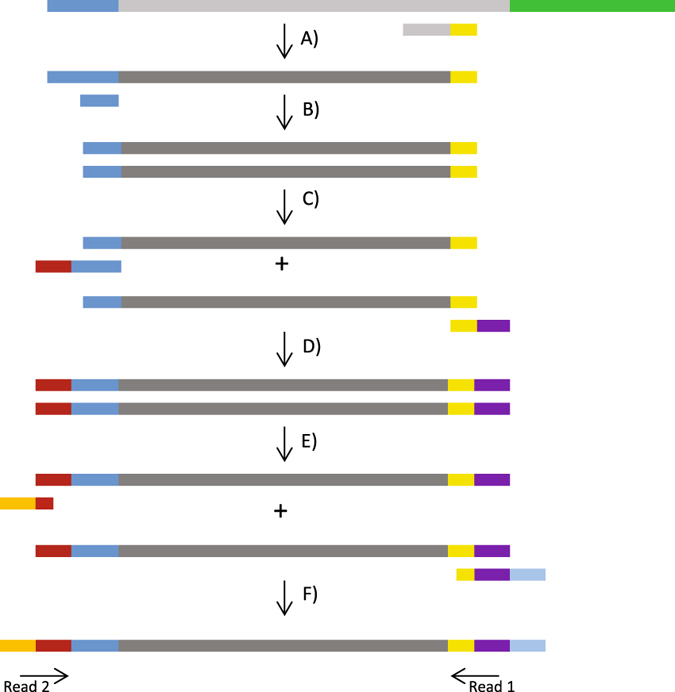
Graphical summary of the SL-seq protocol. (A) cDNA generation with Superscript III and a primer that is partially random (7 nucleotides, grey), and partially fixed (yellow). Consequent degradation of the RNA strand with RNAse H, leaving a single stranded DNA molecule. (B) Second strand synthesis of SL-containing DNA molecules with Klenow fragment and a primer that is complementary with the SL (dark blue). (C + D) PCR for amplification and addition of adapter motives (red and purple) making the library compatible with the Nextera XT index kit (Illumina). (E + F) Final PCR adapter extension, indexing (orange and light blue) and amplification of the library fragments with the primers of the Nextera XT index kit. Dark Blue: SL/ complementary with SL, light grey: RNA, dark grey: DNA, green: poly-A tail. Other colors: primer and adapters sequences.

The protocol that we describe here (Fig. 1) is built upon the existing SL-trapping protocols^7–9^ and the Illumina 16 S Metagenomic Sequencing protocol. A working protocol can be found in Suppl. Material S1.

#### cDNA synthesis

cDNA was synthesized with the Superscript III kit (ThermoFisher Scientific) according to the manufacturer's protocol and using 1 µg of total RNA. Primer 5′-GTATAAGAGACAGNNNNNNN-3′* was added to a final concentration of 2 µM. The RNA strand was degraded by adding 2U of RNAse H (Life Technologies) and 20 minutes incubation at 37 °C. The DNA strand was purified with Agencourt AMPure XP beads (Beckman Coulter). In the complete protocol, these beads are used following the guidelines in the Illumina 16 S metagenomics library prep kit manual.

#### Second DNA strand synthesis

The second strand was generated while enriching for DNA fragments containing the mRNA specific SL sequence. This selection was carried out by using primer 5′-TCAGTTTCTGTA-3′, complementary to the *L. donovani* SL. Some other Trypanosomatid species have slightly different SLs and therefore the SL primer has to be adapted accordingly. A blast search on the *L. donovani* SL showed that no adaptation of the protocol is needed for *L. infantum, L. braziliensis, L.mexicana, L. major, L. amazonesis, L. tarentolae, L. infantum* and many *Crithidia* and *Leptomonas* species, because of 100% identity of the SL (Suppl. Material S2). For several pathogenic Trypanosomes, the SL sequences are described in Gonzalez-Andrade *et al*.^15^.

Second strand synthesis was performed by first incubating 1x NEB buffer, 0.6 mM SL primer and 25 µL of DNA from the previous step at 98 °C for 5 minutes. The mixture was allowed to slowly cool down to room temperature over a period of 30 minutes allowing the primers to anneal. Finally, 5U Klenow fragment and 0.4 mM dNTPs were added to a final reaction volume of 50 µL and the reaction was incubated at 37 °C for 60 minutes. The dsDNA was purified with AMPure XP beads.

#### Addition of Illumina adapters, amplification and sample indexing

Additional adapter sequences were added on 5′ and 3′ end of the library fragments to make them compatible with the Nextera XT Index Kit (Illumina). Both the primer sequences and the protocol of this step are largely based on the Illumina metagenomics library prep protocol. The HiFI Hotstart Ready Mix (KAPA) was used with 2.5 µL of dsDNA from the previous step and 0.04 µM of primers 5′*-GTCTCGTGGGCTCGGAGATGTGTATAAGAGACAG* *ATCAGTTTCTGTACTTTA* *-*3′* and 5′-TCGTCGGCAGCGTCAGATGTGTATAAGAGACAG*. The underlined part of primer 1 corresponds to the SL specific section that should be adapted for organisms with a different SL. This PCR mix was incubated in a thermocyler with following program: denaturation 95 °C −5 min, 18 cycles of denaturation 98 °C −20 s + annealing 55 °C −30 s + extension 72 °C −60 s, and a final extension for 5 minutes at 72 °C. The library fragments were purified with AMPure XP beads.

Finally, the indices from the Nextera XT kit were used to add unique barcodes to each sample. The sequences of these primers have also been released by Illumina (http://support.illumina.com/downloads/illumina-customer-sequence-letter.html). The barcodes were added and the fragments amplified with the KAPA HiFi Hotstart Readymix combined with 5 µL of primer i5 and i7. This was followed by PCR 95 °C for 3 minutes, 8 cycles of denaturation 98 °C −20 s + annealing 55 °C −30 s + 72 °C −60 s, 72 °C for 5 minutes. The final library was purified with AMPure XP beads.

*Oligonucleotide sequences© 2016 Illumina, Inc. All rights reserved. Derivative works created by Illumina customers are authorized for use with Illumina instruments and products only. All other uses are strictly prohibited.

### Read processing and differential expression analysis

#### RNA-seq data from axenic promastigotes

The quality of the sequencing reads was verified with FastQC v0.11.4^16^. Reads were subsequently quality trimmed using Trimmomatic v0.35^17^. For paired-end SL-seq libraries, an additional trimming step was performed: the first 21 bases of the second read were hard clipped since they are part of the SL sequence and do not map to the gene locus to which the sequenced mRNA corresponds. Reads were subsequently aligned with BWA mem 0.7.15^18^ to the *Leishmania donovani* LdBPKv2 reference genome using standard mapping parameters. A conversion table to LdBPKv1 is supplied in Suppl. Dataset S1. BWA mem does not take mRNA (cis-)splicing into account, which does not pose a problem since this process is absent in *Trypanosomatids*. SAM-BAM conversions and mapping statistics were performed with samtools 1.3.1^19^. Count tables were generated using HTseq^20^. HTseq required a gene feature format (gff) annotation file and for Illumina mRNAseq data, the standard CDS annotation provided with the reference genome was used. However, reads obtained with our SL-seq protocol often do not align in the CDS but in the 5′ UTR, since this is the location where the SL is spliced onto the mRNA. Therefore a conventional annotation file could not be used. To convert this annotation file to a suitable format we developed a conversion tool. Briefly, this tool converts the user provided annotations and allocates the non-coding genomic region just upstream to the respective gene. The full SL-seq bioinformatic workflow and the gff conversion tool are available at GitHub in the SL-seq repository (https://github.com/CuypersBart/SL-Seq).

#### RNA-seq data from amastigotes in infected macrophages and human RNA mixes

The analysis was performed identically as described in section ‘RNA-seq data from axenic promastigotes’ with one exception. Trimmed reads were first aligned with TopHat2^21^ to the human genome (hg38) to filter out reads originating from human macrophage RNA. Unmapped reads were then converted back to FASTQ files with samtools 1.3.1 for further analysis with the standard pipeline.

### Statistical and Gene Ontology Analysis

Differential expression analysis was performed with the R package DESeq2^22^. Briefly, DESeq2 generated three values for each gene that were used for subsequent analysis: 1) the log2 fold change (Log2FC), i.e. the log2 ratio of gene expression between two parasite life stages, 2) *p*-values based on the Wald test, and 3) corrected *p*-values controlling the false discovery rate to 5%. Genes were considered differentially expressed if the corrected *p*–value was lower than 0.05. For the biological interpretation of the results, we placed an additional log2FC cut-off of at least 1 for upregulation, or −1 for downregulation. To compare the median normalized read counts and the log2FCs obtained from the SL library with those obtained from the Illumina library, regression analysis was performed in R. Plots were generated using the R package ggplot2^23^.

The complete pipeline from raw sequencing reads to statistical analysis was repeated for subsets of the raw sequencing files (0.25, 0.5, 1, 2, 2.5 million reads per sample) to determine which amount of sequencing reads is sufficient to detect most genes, most differentially expressed genes, and to check which of both sequencing methods yields the best performance.

Gene Ontology (GO) enrichment analysis was used to determine which functional gene categories were upregulated or downregulated in the different *Leishmania* life stages. This analysis was performed in Cytoscape^24^ using the BiNGO plugin^25^. The input data consisted of a list of either up- (adjusted *p*-val < 0.05, log2FC > 1) or down-regulated genes (adjusted *p*-val < 0.05, log2FC < −1). BiNGO uses a hypergeometric test to calculate enrichment *p-*values and we corrected for multiple testing with Benjamini-Hochberg (FDR < 0.05). Since this approach still relies on the choice of specific cut-offs for log2FC and *p*-value, we also used the cut-off free Gene Set Enrichment Analysis (GSEA) on the lists of log2FC of all genes. GSEA was performed using the GSEAPreranked tool version 4.5, available on the GenePattern webserver of the Broad Institute^26^. Gene sets were considered significantly changed if the family wise error rate corrected *p*-value was lower than 0.05. Venn diagrams were generated to show the overlap between the GO enrichment analysis or GSEA and were generated with the UGent Bioinformatics & Evolutionary Genomics group web tool (http://bioinformatics.psb.ugent.be/webtools/Venn/).

## Results

### Technical and functional comparison of SL-seq and Illumina RNA-seq

**Figure 2:**
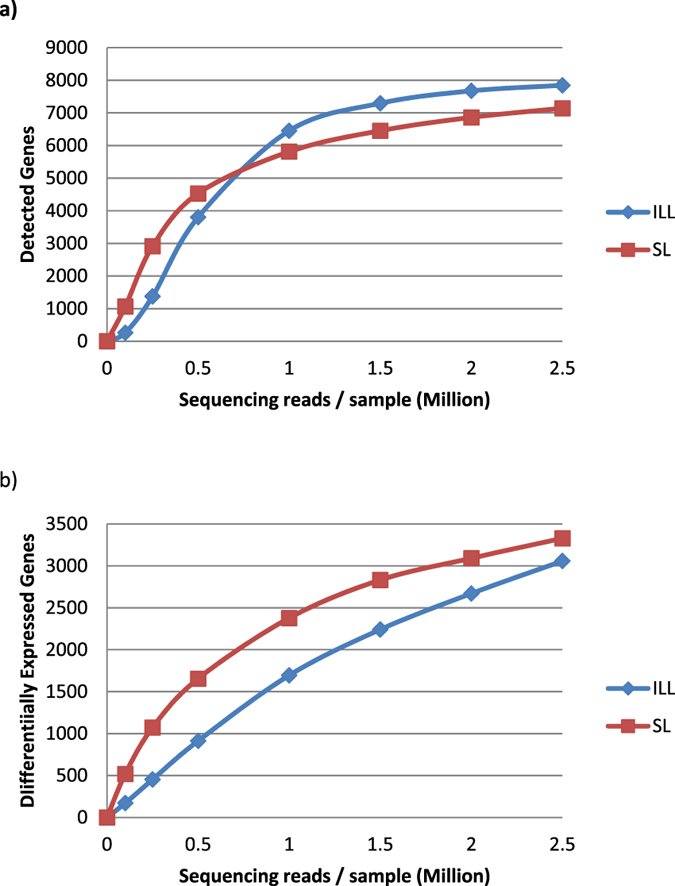
(**a**) Relation between the raw sequencing depth per sample and the amount of detected genes. Genes were considered detected if they had at least 10 counts in every sample. Every point represents a full differential expression analysis started from a specified amount of raw sequencing reads, sampled from the original files. (**b**) Relation between the raw sequencing depth per sample and the amount of detected differentially expressed genes (DESEQ2 FDR Adjusted *p* < 0.05) between LOG and STAT *Leishmania* promastigotes.

**Figure 3:**
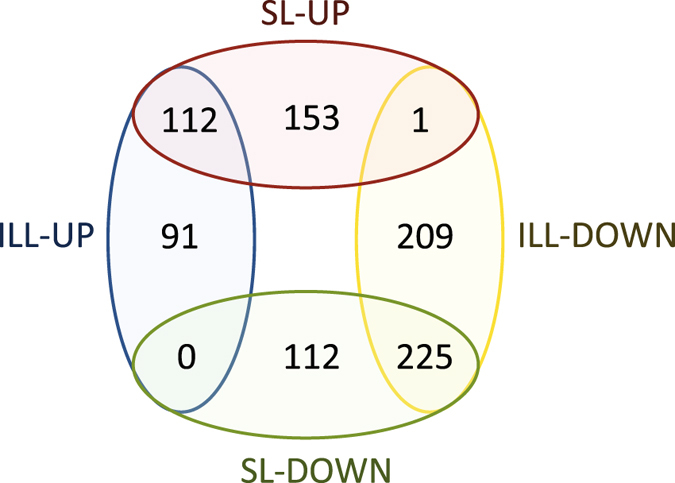
Differentially expressed genes obtained with SL-seq (SL-UP and SL-DOWN) and Illumina stranded mRNA seq methods (ILL-UP and ILL-DOWN). Genes were considered significantly upregulated if the FDR adjusted *p-*value was lower than 0.05 and the log2FC greater than 1. Genes were considered significantly downregulated if the FDR adjusted *p-*value was lower than 0.05 and the log2FC bigger smaller than −1.

We compared the results of SL-seq with those obtained with ILL-seq on exactly the same mRNA. Pure RNA of quadruplicate *L. donovani* BPK275 promastigote cultures was extracted at both the logarithmic and stationary phases and sequenced with both the Illumina stranded mRNA and the SL-seq protocols. The sequencing statistics, mapping statistics, count tables and the output from DESEQ2 can be found in Suppl. Dataset S2. In total, we detected 8234 genes with ILL-seq of which 4862 were differentially expressed between LOG and STAT promastigotes, while this was also the case for 4191 of the 7544 genes detected with SL-Seq. However, SL-Seq detected more genes than ILL-seq when the sequencing coverage for each sample was sampled below 0.75 million reads (Fig. 2a). This difference is the largest around a sequencing coverage of 0.25 million reads per samples, where SL-seq detects 2.4 times more differentially expressed genes than ILL-seq. Above this threshold, ILL-seq detects more transcripts, but only 1.1 times at 2.5 million reads per sample. Interestingly, SL-seq always finds more differentially expressed genes than ILL-seq, when sequencing both libraries at the same depth (Fig. 2b). This difference is more pronounced at low sequencing coverage. For example, when sequencing 0.5 million reads per biological replicate, SL-seq detected 1655 differentially expressed genes (DEGs), which is 82% more than the 913 detected by ILL-seq. At 2.5 million reads per replicate we found 3338 DEGs for SL-seq and 3058 (9% less) for ILL-seq. Although in this study 16% more DEGs were observed for the Illumina stranded mRNA seq than SL-seq, this can be fully explained by the fact that the Illumina libraries were sequenced deeper. Many of these DEGs exhibited only minor log2 fold changes (log2FC) and therefore a minimal log2FC of 1 was applied to select the biologically most relevant genes. With the Illumina kit, 203 genes were found to be significantly upregulated (FDR corrected *p-*val < 0.05, log2FC > 1) in STAT, while 435 were downregulated (FDR corrected *p-*val < 0.05, log2FC < −1). SL-seq revealed 265 upregulated and 337 downregulated genes. The overlap between the two datasets is shown in Fig. 3. Generally, genes found to be differentially up- or downregulated with SL-seq showed the same change in the ILL-seq, or none at all, and vice versa. A large proportion (57%) of the genes upregulated in SL-Seq did not show a significant up- or downregulation in ILL-seq (FDR corrected *p-*val < 0.05, |log2FC| < −1), as was also the case for 33% of the genes downregulated in SL-Seq. However, when these genes were examined more closely (Suppl. Dataset S3) most of them showed a significant change in the identical direction, but with a lower log2FC or without statistical significance. This strongly suggests that SL-Seq and ILL-Seq largely do report the same biological information, but might have slightly different statistical power or different biases for individual genes. In addition, the obtained log2FCs are still an estimates for both techniques and due to random technical variation genes might or might not just reach the threshold. More specifically, an additional 95 genes upregulated in SL-seq, showed also upregulation in ILL-seq, when the log2FC cut-off was lowered to 0.5 (FDR corrected *p-*val < 0.05, 1 > log2FC > 0.5). 46 genes still did not exhibit a change (*p*-val > 0.05 and/or |log2FC| < 0.5), and 6 were downregulated (FDR corrected *p-*val < 0.05, log2FC < −0.5). Similarly, 79 of 112 genes that were unchanged in ILL-seq, but downregulated in SL-seq, also became downregulated in ILL-seq when lowering the log2FC threshold to −0.5, while 30 did not change and 3 were not detected. In summary, this means that 85% of the genes that were significantly 2 fold up- or downregulated in SL-seq (*FDR corrected p-val < 0.05*), also exhibited a significant and corresponding change in ILL-seq (*FDR corrected p-val < 0.05*), |log2FC| > 0.5).

**Figure 4:**
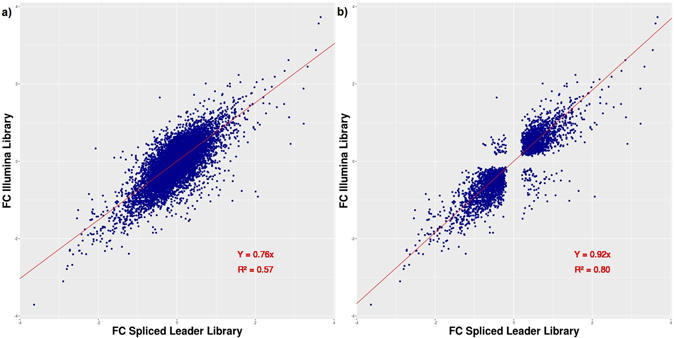
Correlation between log2 fold change obtained from sequencing with SL-seq versus Illumina stranded mRNA library prep. (**a**) All genes are plotted for which counts were obtained in both library preps. (**b**) Only genes plotted that had a significant LOG-STAT fold change (DESEQ2 adjusted *p* < 0.05) with both SL-Seq and ILL-Seq.

The significance cutoff for differential expression may result in several genes found with only one method, while the difference in the log2FC values between SL-seq and ILL-seq could actually be very small. We therefore performed a linear regression of the log2FCs obtained with these two methods (Fig. 4a). Although, the correlation was highly significant (*p* < 2e-16), the R² of only 0.57 shows a low predictive power. This improved considerably when we excluded all genes with a non-significant log2FC in either of the two libraries (Fig. 4b). In this manner, genes that show only random variation among the biological replicates, even within the same life stage, were removed. The R² then increased to 0.92 and the slope to 0.80, which indicates that the significant log2FCs obtained with either method are very similar. Linear regressions between SL-Seq and ILL-Seq were also generated using the read counts directly (only normalized for sequencing depth) for both LOG and STAT life stages (Suppl. Material S3). Although this correlation was highly significant in both cases (p < 10^−16^), the predictive value was very low (R² = 0.11 for LOG and R² = 0.09 for STAT). This difference in measured RNA abundance is in line with expectations as large differences are commonly observed when using different library prep methods^27^. These differences in raw expression values are likely caused by different enrichment and PCR amplification biases. However, despite these differences, the relative abundance as denoted by the fold changes is much less impacted.

**Figure 5:**
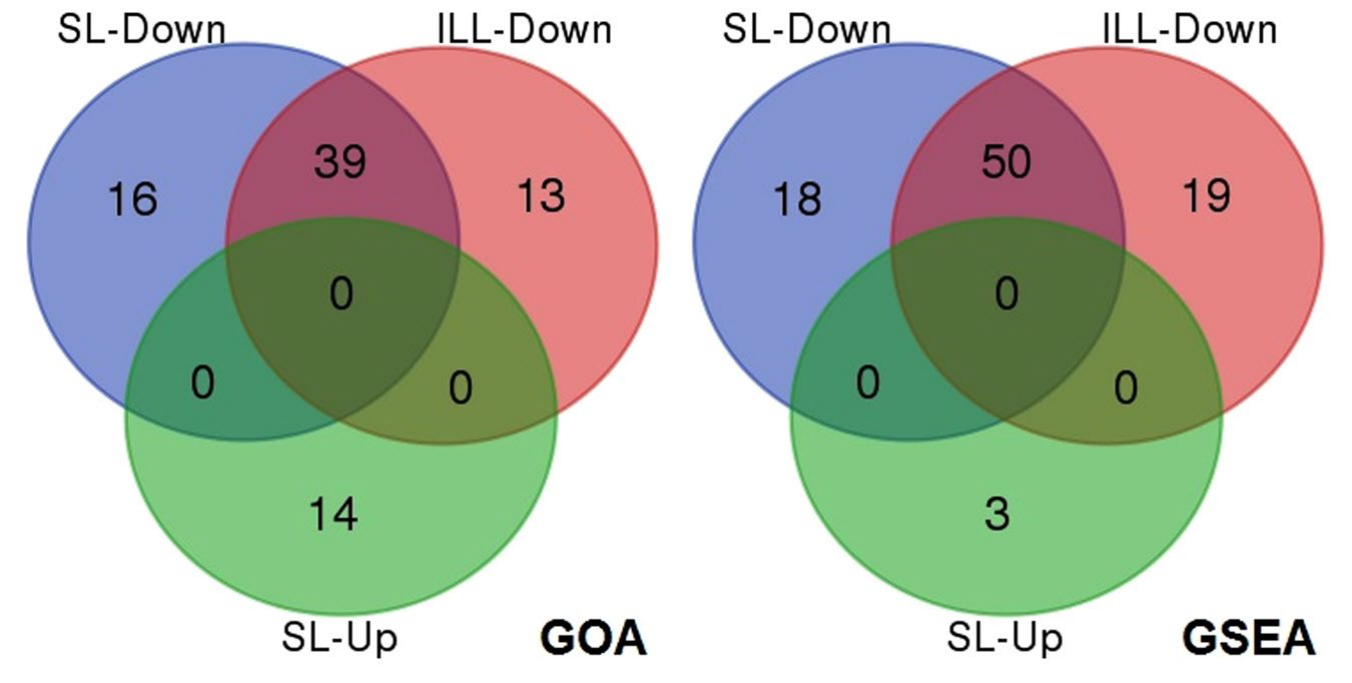
Venn diagrams showing the overlap between SL-Seq or ILL-Seq of GO sets found downregulated (Down) or upregulated (Up) with GOA (left) and GSEA (right). Note: For both methods, no upregulated GO sets were found for ILL-Seq.

We performed GO enrichment analysis (GOA) to investigate the main biological changes between LOG and STAT promastigotes, for both the SL-seq and ILL-seq libraries. We provide here a summary of the most relevant results, but the full analysis is available in Supplementary Material S4, S5 and Suppl. Dataset S4. We observed a major overlap in functional results between the two methods (Fig. 5). Both SL-seq and ILL-seq showed a significant decrease in ATP-synthesis related transcripts in STAT. GOA indicated that several components of the mitochondrial ATP-synthase complex were differentially expressed, more specifically the subunit beta, delta, epsilon and c were downregulated in both SL-seq and ILL-seq, while additionally, the oligomycin sensitivity conferral protein (OSCP) subunit was downregulated in SL-seq and the gamma subunit in ILL-seq. Notably, ATP-synthase gamma was also significantly downregulated in SL-seq, but with a log2FC of just −0.94. The same was true for the OSCP unit in ILL-seq with a significant fold change of −0.95. Transmembrane proton transport was a second biological process that was significantly downregulated in STAT in both SL-seq and ILL-seq. Apart from the described changes in ATP synthase, we observed decreases in the mRNA of V-ATPase subunit c, subunit b and subunit e as well as a vacuolar-type proton translocating pyrophosphatase 1. Oxidoreductase activity with NAD or NADP as electron donor was also significantly reduced according to GOA. Respectively 8 and 9 NADH generating enzymes were downregulated in SL-seq and ILL-seq including both isocitrate and malate hydrogenase. Further, GOA highlighted a decrease of sequence specific DNA binding gene transcripts in STAT: downregulations were observed for 3 histone H4 genes and 2 histone H3 genes in both ILL-seq and SL-seq. Also lower levels of the core histone-like transcription factor (CBF/NF-Y) were detected in ILL-seq and a histon H4 in SL-seq. Some GO terms were exclusively enriched with SL-seq where a significant upregulation was detected of genes related to post-translational protein modifications, more specifically, protein phosphorylation by mRNA increases of several protein kinases. Many of these kinases were however also significantly upregulated in ILL-seq (Suppl. Material S5). GO enrichments detected exclusively in ILL-seq included downregulation of transcription elongation including the genes eukaryotic initiation factor 5a, elongation factor1-beta, translation elongation factor 1 beta and elongation factor 1 gamma. Furthermore, in ILL-seq, GOA indicated significant decreases in protein folding capacity, by downregulation of mRNA for HSP90, HSP10, cyclophilin 40, cyclophilin-type peptidyl-prolyl cis-trans isomerase, co-chaperone GrpE, Peptidyl-prolyl cis-trans isomerase, prefoldin subunit 2 and calreticulin. For both GO classes, several of these genes were also significantly downregulated in SL-seq **(**Suppl. Material S5 **)**.

The GOA performed in Cytoscape required us to select genes for the test-sets, for which we used the criteria adj. *p* < 0.05 and |log2FC| > 1. Although this is an excellent approach when focusing on the most heavily affected genes or functions, it might fail to highlight GO terms for which the genes changed in a lesser extent, just not reaching the stringent thresholds we defined. Therefore we also used a complementary analysis technique called Gene Set Enrichment Analysis (GSEA) using the GO terms as gene sets, and compared the results between SL-seq and ILL-seq. GSEA does not require any *p*-value or Log2FC cut-off, but ranks the genes according to their Log2FC and checks if there are significantly more genes of a specific GO term towards the top or bottom of the list. GSEA yielded similar results as GOA (Suppl. Dataset S5). Briefly, 68 GO categories were downregulated in STAT for SL-seq and 69 for ILL-seq, of which 50 were shared between both (Fig. 5). Just like the GOA results, the shared categories were mostly related to proton transport and ATP-synthesis, oxidoreductase activity with NAD or NADP as electron donor. Furthermore, a downregulation of translation related processes in STAT was observed with both methods, but this was evidenced by translation factor GO terms for SL-seq, while for ILL-Seq GO sets related to t-RNA synthesis were enriched. Only 3 GO sets were found upregulated in STAT for SL-seq (protein kinase activity, protein phosphorylation and phosphorylation), while none were found for ILL-Seq.

### Relative enrichment of *Leishmania* mRNA over human mRNA with SL-Seq

To assess how well SL-seq performs in the presence of high relative concentrations of human RNA, we performed this protocol on 2 dilutions at a *Leishmania*:human RNA ratio of 1:100, 1:1000 and undiluted control samples (Table 1). For the undiluted samples, on average 96.38% of the reads could be mapped to the *L. donovani* genome, while this was 57.69% for the 1:100 dilution and 12.44% at 1:1000. Aspecific amplification of human DNA increased accordingly from 0.18% in the undiluted sample to 76.40% in the highest dilution. However, this shows that although the *Leishmania* RNA was diluted with 1000 x more human RNA, this resulted only in a 7-fold drop of SL-seq data.

**Table 1:** Proportion of reads mapping to respectively human and *Leishmania* genomes in control conditions and in samples diluted 1/100 and 1/1000 with human RNA.

Dilution	Human		*Leishmania*		Theoretical enrichment
	Average(%)	St.Dev	Average(%)	St.Dev	
0	0.18	0.05	96.38	0.58	NA
1/100	35.21	1.50	57.69	2.40	59.86x
1/1000	76.40	0.88	12.44	0.96	129.07x

The last column indicates how many times SL-seq enriched the *Leishmania* mRNA compared to the Illumina kit.

### SL-seq and Illumina sequencing of infected macrophages

We tested the capacity of the SL-seq method to sequence *Leishmania* BPK275 and BPK282 amastigote mRNA directly from infected THP-1 cells without prior parasite purification (Table 2). Non-infected monocytes were included in the experiment as a control. Respectively 60% and 70% of the THP-1 cells contained amastigotes after exposure to BPK282 or BPK275 promastigotes. Cells infected with BPK282 or BPK275 respectively contained on average 3.4 or 4 parasites. All samples were also sequenced with the Illumina stranded mRNA kit to determine the enrichment of *Leishmania* mRNA with SL-seq. On average 64.95% of the reads mapped to the *L*. *donovani* reference genome using SL-Seq, while 24.25% mapped to the HG38 genome. This indicates aspecific amplification occurs in the presence of non-*Leishmania* mRNA. These aspecific sequences were not restricted to a specific region, but originated from all over the human genome. However, when the same RNA was sequenced with ILL-seq, only 1.58% of the reads mapped to the *L. donovani* reference genome and 96.64% to HG38, indicating SL-Seq results in a relative 44.82 fold enrichment of *Leishmania* mRNA sequence data.

**Table 2:** Relative enrichment of *Leishmania* mRNA with SL-sequencing from infected THP-1 monocytes.

Sample	Infection	Illumina Stranded mRNA		SL-Seq		Enrichment (X)
		%Human	%*Leishmania*	%Human	%*Leishmania*	
1	NI	98.14	0.06	84.16	0.55	
2	NI	98.09	0.06	84.60	0.47	
3	BPK275	97.09	1.17	28.89	64.75	55.17
4	BPK275	96.85	1.36	29.13	64.89	47.61
5	BPK282	96.31	1.89	23.38	70.92	37.44
6	BPK282	96.31	1.90	20.73	74.29	39.06
					Average	44.82 ± 8.2

THP-1 monocytes infected with *Leishmania* strain BPK275 and BPK282 and uninfected controls were sequenced with both SL-seq and a conventional poly-A mRNA kit (Illumina stranded mRNA). Displayed percentages show the relative amount of sequencing reads mapping to the human (hg38) and *Leishmania* genome with each kit. The last column indicates how many times SL-seq enriched the *Leishmania* mRNA compared to the Illumina kit.

Since the macrophages infected with BPK275 and BPK282 had similar mapping percentages as the 1:100 dilution, we estimate that SL-seq is also able to profile the transcriptome of infections that contain even 10 x less parasites (cfr. the 1:1000 dilution) than in our setup.

We compared the expression profiles of amastigotes obtained from SL-seq of infected THP-1 with those of STAT promastigotes. Respectively 588 genes were significantly upregulated (FDR corrected *p-*val < 0.05, log2FC > 1) in amastigotes, while 624 genes were significantly downregulated (FDR corrected *p-*val < 0.05, log2FC < -1) (Suppl. Dataset S2). GOA and GSEA showed that the downregulated transcripts were significantly enriched for genes related to RNA binding, translation and ribosome, and more specifically constituents of ribosomes (Suppl. Material S4, Suppl. Dataset S4, S5). Indeed, we observed a striking decrease of genes coding for ribosomal components in amastigotes, where 72 were significantly downregulated, 20 of them exhibiting extreme log2FCs in the range of −4 to −8. The upregulated genes did not show any significantly enriched GO terms.

## Discussion

In this work, we presented and validated a mRNA profiling method called SL-seq that we developed to sequence Trypanosomatid transcriptomes directly from the infected host cells in a high-resolution and high-throughput fashion.

We first analyzed whether the transcriptome profiles obtained with SL-seq were similar to those obtained with ILL-seq. A match could be expected since mature mRNA contains both a SL and a polyA-tail, targeted by SL-seq and ILL-seq respectively. However, the SL and poly A-tail are not added at exactly the same time to the final monocistronic transcript. Indeed, since trans-splicing and polyadenylation are linked processes, polyadenylation at the 3′ end of the downstream gene occurs when the SL is added to the 5′ end of the upstream gene^28^. In addition, at the technical level, there could also be different library prep and sequencing biases between SL-seq and ILL-seq. To compare these two sequencing methods, we analyzed the gene expression profiles of *L. donovani* promastigotes from LOG and STAT phases using both techniques. We found a strong overall correlation between these fold changes with considerable predictive value between both methods when focusing on the significant genes. These observations clearly indicate that SL-seq and ILL-seq enumerate largely the same pool of mRNA. This overlap was further evaluated at the biological level. GOA and GSEA clearly pointed out that SL-seq and ILL-seq detected predominantly the same biological changes between LOG and STAT promastigotes. LOG phase cultures mainly contain rapidly dividing, procyclic promastigotes, while STAT phase cultures contain infective, metacyclic promastigotes. Both SL-seq and ILL-seq detected molecular changes consistent with this differentiation. We observed a clear downregulation of ATP-synthesis in STAT phase parasites which is in agreement with a lower metabolic rate and therefore ATP requirement, as has previously been observed for *L. major* ^6^. More specifically, STAT phase parasites had decreased mRNA levels of several ATP-synthase components, including subunits beta, delta, epsilon and c. Decreased expression was also detected for several genes involved in NADH production, including 2 key enzymes of the Krebs cycle: isocitrate and malate hydrogenase. Since NADH is the main ‘input fuel’ for the oxidative phosphorylation chain, this is also consistent with the downregulation of ATP-synthesis in metacyclics. Interestingly, these results show that the diminished energy production in metacyclics is the result of a coordinated action at many different points in the energy metabolism, rather than just one individual bottleneck, evidencing a fine-tuned and complex mRNA regulation. According to ILL-seq, protein folding and transcription elongation factors exhibited lower expression in STAT phase parasites, probably linked to the lower metabolic state. Also translation related processes showed downregulation with both SL-seq and ILL-seq. While procyclic promastigotes have active DNA replication and cell division, these processes are downregulated in metacyclics. Correspondingly, both SL-Seq and ILL-seq demonstrated significant reductions in mRNA coding for DNA binding proteins like histone H3 and H4, which are typically needed during DNA replication^29^. Finally, SL-seq showed a strong upregulation of protein kinases in STAT phase promastigotes, which modulate the activity of other proteins. This increase in kinase activity during metacyclogenesis has been observed before and plays a major role in infectivity by phosphorylation of complement proteins, thus attracting the host cells^6, 30^. In summary, SL-seq and ILL-seq show largely the same technical and biological results, aligning well with what is known from other studies. We therefore conclude that the little differences we observed are most likely caused by technical variation, while biologically both methods enumerate largely the same pool of mRNA. Future studies are required to check if these small differences are indeed technical variation or the result of the underlying biological mechanism of transcription.

A second part of this work consisted in the technical benchmarking of our pipeline. The consideration of how many reads to generate per sequencing library is one of the most important ones in the experiment. Not only does it determine the statistical power with which DEGs will be detected, it is also one of the main, if not the most, contributing factors to the final cost of the experiment. Therefore we determined the effect of sequencing depth on the number of detected DEGs, for both ILL-seq and SL-seq libraries. Interestingly, we observed that for SL-seq with 4 biological replicates and 2 conditions, 1 million reads for each replicate is already sufficient to find approximately half of all DEGs. Based on these results, we recommend to sequence 3 million reads per SL-seq library, when working with 4 biological replicates. Furthermore, SL-seq required less sequencing depth to find the same number of DEGs compared to ILL-seq.

Many SL-containing organisms are pathogens and are most relevant to study when residing within their biological host, rather than isolated in cell culture. For RNA-seq studies attempting to characterize the RNA profile of the pathogen this imposes the problem that host RNA is often much more abundant. Indeed, in this study, we found that with ILL-Seq only 1.58% of the RNAseq data originated from *Leishmania* when sequencing directly from infected THP-1 cells. Therefore, obtaining sufficient reads for an in-depth analysis of the transcriptome would require either very deep sequencing per sample, which is costly, or prior extraction which is labor intensive and requires a relatively high quantity of starting material^31^. In addition, extraction of parasite cells from the host increases the chance that experimental and environmental factors affect the transcriptome. Indeed, *Leishmania* is adapted to quickly respond to a different environment and can for example revert from the amastigote to the promastigote life stage within 24–48 hours^32^. We demonstrated that SL-seq completely abolishes the need for prior extraction or deep sequencing. The protocol is fast, simple and enriches the *Leishmania*:human mRNA ratio around 44-fold. This makes SL-seq the ideal method to profile the transcriptomes of parasites and other symbionts. To study host-pathogen interactions, SL-Seq can even be applied in parallel with conventional RNAseq on the same RNA extract to profile the transcriptome of both parasite and host. The results obtained with SL-seq from amastigotes nicely correspond to what is known about amastigotes in the literature. We observed striking decreases in genes related to translation and ribosome structure, consistent with the quiescent status of amastigotes and the observed lower protein turnover^33^.

Finally, we showed that SL-seq can be used for samples with a very low (1:1000) *Leishmania*:human RNA ratio. Although these samples will have to be sequenced deeper (7 times for 1:1000) than samples containing pure *Leishmania* RNA to obtain the same coverage, this high sensitivity and specificity are very promising for clinical applications. For example, sequencing parasites directly from patient tissue samples without prior parasite isolation and cultivation could increase both the speed and the relevance of studies on clinical samples. The combined multiplexing capacity and simple PCR-based steps of SL-seq allow to easily prepare and sequence hundreds of samples in parallel.

In summary, we presented and validated a new, multiplexed SL-seq protocol for high throughput, selective RNA-seq in *Leishmania*. We have successfully applied this method to sequence *Leishmania* mRNA directly from infected macrophages and from highly diluted mixes with human RNA. We showed the obtained results are able to capture the biology of the parasite and benchmarked our method against a conventional mRNA-seq method. SL-seq is therefore ideal for fast, accurate and massive parallel sequencing of parasite transcriptomes directly from the host tissue. Additionally, the high resolution splice-site mapping could offer major advantages for future studies of mRNA regulation. Since the presence of a SL and trans-splicing are shared with many other phylogenetic groups including trypanosomess, nematodes, trematodes and primitive chordates, this method has also high potential for *in vivo* studies of other symbionts.

## Electronic supplementary material

Supplementary Material

Supplementary Dataset 1

Supplementary Dataset 2

Supplementary Dataset 3

Supplementary Dataset 4

Supplementary Dataset 5

## References

[1] Alvar J (2012). Leishmaniasis Worldwide and Global Estimates of Its Incidence. PloS one.

[2] Vanaerschot M (2014). Treatment failure in leishmaniasis: drug-resistance or another (epi-) phenotype?. Expert review of anti-infective therapy.

[3] Martinez-Calvillo S, Nguyen D, Stuart K, Myler PJ (2004). Transcription initiation and termination on Leishmania major chromosome 3. Eukaryotic cell.

[4] Günzl, A. in *RNA Metabolism in* Trypanosomes (ed. Albrecht Bindereif) 1–27 (Springer Berlin Heidelberg, 2012).

[5] Preußer, C., Jaé, N., Günzl, A. & Bindereif, A. in *RNA Metabolism in* Trypanosomes (ed. Albrecht Bindereif) 49–77 (Springer Berlin Heidelberg, 2012).

[6] Dillon LAL (2015). Transcriptomic profiling of gene expression and RNA processing during Leishmania major differentiation. Nucleic acids research.

[7] Nilsson D (2010). Spliced Leader Trapping Reveals Widespread Alternative Splicing Patterns in the Highly Dynamic Transcriptome of Trypanosoma brucei. PLOS Pathogens.

[8] Haydock A (2015). RNA-seq approaches for determining mRNA abundance in Leishmania. Methods in molecular biology (Clifton, N.J.).

[9] Mittra B (2013). Iron uptake controls the generation of Leishmania infective forms through regulation of ROS levels. The Journal of experimental medicine.

[10] Kolev NG, Ullu E, Tschudi C (2015). Construction of Trypanosoma brucei Illumina RNA-Seq libraries enriched for transcript ends. Methods in molecular biology (Clifton, N.J.).

[11] Aslett M (2010). TriTrypDB: a functional genomic resource for the Trypanosomatidae. Nucleic acids research.

[12] Rastrojo A (2013). The transcriptome of Leishmania major in the axenic promastigote stage: transcript annotation and relative expression levels by RNA-seq. BMC Genomics.

[13] Mourão MdM (2013). A directed approach for the identification of transcripts harbouring the spliced leader sequence and the effect of trans-splicing knockdown in Schistosoma mansoni. Memórias do Instituto Oswaldo Cruz.

[14] Imamura, H. *et al*. Evolutionary genomics of epidemic visceral leishmaniasis in the Indian subcontinent. *eLife* **5**, doi:10.7554/eLife.12613 (2016). 10.7554/eLife.12613 PMC4811772 27003289

[15] Gonzalez-Andrade P (2014). Diagnosis of trypanosomatid infections: targeting the spliced leader RNA. The Journal of molecular diagnostics: JMD.

[16] Andrews, S. FastQC: a quality control tool for high throughput sequence data. (2010).

[17] Bolger AM, Lohse M, Usadel B (2014). Trimmomatic: a flexible trimmer for Illumina sequence data. Bioinformatics (Oxford, England).

[18] Li, H. Aligning sequence reads, clone sequences and assembly contigs with BWA-MEM. *arXiv preprint arXiv:1303.3997* (2013).

[19] Li H (2011). A statistical framework for SNP calling, mutation discovery, association mapping and population genetical parameter estimation from sequencing data. Bioinformatics (Oxford, England).

[20] Anders S, Pyl PT, Huber W (2015). HTSeq-a Python framework to work with high-throughput sequencing data. Bioinformatics (Oxford, England).

[21] Kim D (2013). TopHat2: accurate alignment of transcriptomes in the presence of insertions, deletions and gene fusions. Genome biology.

[22] Love MI, Huber W, Anders S (2014). Moderated estimation of fold change and dispersion for RNA-seq data with DESeq2. Genome biology.

[23] Wickham, H. *ggplot2: elegant graphics for data analysis*. (Springer Science & Business Media, 2009).

[24] Shannon P (2003). Cytoscape: a software environment for integrated models of biomolecular interaction networks. Genome research.

[25] Maere S, Heymans K, Kuiper M (2005). BiNGO: a Cytoscape plugin to assess overrepresentation of gene ontology categories in biological networks. Bioinformatics (Oxford, England).

[26] Subramanian A (2005). Gene set enrichment analysis: a knowledge-based approach for interpreting genome-wide expression profiles. Proceedings of the National Academy of Sciences of the United States of America.

[27] A comprehensive assessment of RNA-seq accuracy (2014). reproducibility and information content by the Sequencing Quality Control Consortium. Nature biotechnology.

[28] Liang X-h, Haritan A, Uliel S, Michaeli S (2003). trans and cis Splicing in Trypanosomatids: Mechanism, Factors, and Regulation. Eukaryotic cell.

[29] Annunziato AT (2005). Split decision: what happens to nucleosomes during DNA replication?. The Journal of biological chemistry.

[30] Gupta G, Oghumu S, Satoskar AR (2013). Mechanisms of Immune Evasion in Leishmaniasis. Advances in applied microbiology.

[31] Pescher P, Blisnick T, Bastin P, Spath GF (2011). Quantitative proteome profiling informs on phenotypic traits that adapt Leishmania donovani for axenic and intracellular proliferation. Cellular microbiology.

[32] Saar Y (1998). Characterization of developmentally-regulated activities in axenic amastigotes of Leishmania donovani. Molecular and biochemical parasitology.

[33] Kloehn J, Saunders EC, O’Callaghan S, Dagley MJ, McConville MJ (2015). Characterization of Metabolically Quiescent Leishmania Parasites in Murine Lesions Using Heavy Water Labeling. PLOS Pathogens.

