# Supplementary material for: Multiplexed Spliced-Leader Sequencing: A high-throughput, selective method for RNA-seq in Trypanosomatids
Source: Sci Rep. 2017 Jun 16;7:3725. doi: 10.1038/s41598-017-03987-0 (PMC5473914; doi:10.1038/s41598-017-03987-0)
Supplement: Supplementary file 1 — Supplementary Material [file 41598_2017_3987_MOESM1_ESM.pdf]

Multiplexed Spliced-Leader Sequencing: A high-throughput, selective method for RNA-seq in *Trypanosomatids*.

Supplementary Material

Bart Cuypers[1,2], Malgorzata A. Domagalska[1], Pieter Meysman[2], Geraldine de Muylder[1], Manu Vanaerschot[1$], Hideo Imamura[1], Franck Dumetz[1], Thomas Wolf Verdonckt[1], Peter J. Myler[3,4], Gowthaman Ramasamy[3], Kris Laukens[2#] and Jean-Claude Dujardin[1,5*#].

1. Molecular Parasitology Unit, Department of Biomedical Sciences, Institute of Tropical Medicine, Antwerp, Belgium.
2. Advanced Database Research and Modeling group (ADReM), Department of Mathematics and Computer Science, University of Antwerp, Antwerp, Belgium.
3. Center for Infectious Disease Research, Seattle, Washington, United States of America.
4. Department of Global Health and Department of Biomedical Informatics & Medical Education, University of Washington, Seattle, Washington, United States of America.
5. Department of Biomedical Sciences, University of Antwerp, Antwerp, Belgium.

[$] Present address: Fidock Lab, Department of Microbiology, College of Physicians and Surgeons, Columbia University, New York, USA.

#Shared senior authors

* To whom correspondence should be addressed. Tel: 0032 32476355; Email: JCDujardin@itg.be

Supplementary Material Legends (Click to navigate)

Supplementary Material S1: SL-seq working protocol.

Supplementary Material S2: Output of BLAST search with the *Leishmania donovani* SL-sequence to show the similarity with other *Leishmania* and related species.

Supplementary Material S3: Linear regression between SL-Seq and ILL-Seq of the median normalized read counts for logarithmic (a) and stationary phase (b) *Leishmania donovani* promastigotes.

Supplementary Material S4: Hierarchical gene ontology networks from Cytoscape, from the enrichments of Supplementary Dataset S4. Note: empty networks are not shown.

Supplementary Material S5: Gene ontology enrichment analysis summary and comparison between SL-seq and ILL-seq. Genes were considered differentially expressed for a method (SL-Seq/ILL-seq) if $|FC|>1$ and adjusted $p < 0.05$.

Supplementary Datasets (Provided in separate files, not included in this document).

Supplementary Dataset S1: Conversion table from the old (LdBPKv1) to the new the new (LdBPKv2) *Leishmania donovani* reference genome.

Supplementary Dataset S2: The sequencing statistics, mapping statistics, count tables, sequence read archive (SRA) accession numbers and the output from DESEQ2 for all samples in this study.

Supplementary Dataset S3: Overlap between differentially expressed genes in SL-seq and ILL-seq.

Supplementary Dataset S4: Gene ontology enrichments and statistics of the upregulated and downregulated genes in SL-seq and ILL-seq.

Supplementary Dataset S5: Gene ontology sets upregulated (UP) and downregulated (DOWN) according to Gene Set Enrichment Analysis results for ILL-Seq and SL-Seq. Results generated with the GSEAPreranked tool, available on the GenePattern webserver of the Broad Institute.

Supplementary Material S1

cDNA Synthesis

First cDNA strand synthesis

- Add following components to a PCR tube tube:
    - Add 2 µL of 20µM 'strand 1 cDNA primer' (5'- GTATAAGAGACAGNNNNNNN-3') *
    - 1 µg total RNA
    - 2 µL 20mM dNTP mix (5 mM each at neutral pH)
    - Sterile, distilled water to 14µL
- Heat to 65°C for 5 minutes
- Incubate on ice for at least 1 minute
- Centrifuge briefly to collect contents at the bottom of the tube
- Add:
    - 4 µL 5x First strand buffer (Invitrogen)
    - 1 µL 0.1 M DTT
    - 1 µL Superscript III (Invitrogen)
- Mix gently by pipetting up and down
- Incubate at 25°C for 5 minutes
- Incubate for 60 min at 50°C followed by 15 min at 70°C

* Oligonucleotide sequences © 2016 Illumina, Inc. All rights reserved. Derivative works created by Illumina customers are authorized for use with Illumina instruments and products only. All other uses are strictly prohibited. The underlined part

Destroy RNA strand and purify DNA strand

- Add 1µL of 2U RNase H
- Incubate at 37°C for 20 minutes
- Purify using AMPure XP beads (Agencourt)
    - Let the beads adapt to room temperature
    - Vortex Beads
    - Add 37.8 µL of beads (=1.8 * volume reaction mix) and mix by pipetting up and down 10 times
    - Incubate for 10 minutes
    - Place on magnetic stand until the solution is clear
    - Aspirate solution
    - Wash with 200 µL of 80% ethanol (do not disturb the beads)
    - Incubate for 30s
    - Remove ethanol
    - Wash with 200 µL of 80% ethanol (do not disturb the beads)
    - Incubate for 30s
    - Remove ethanol
    - Let the plate dry for 15 minutes

- Add 40 µL of molecular grade water and resuspend the beads
- Incubate for 2 minutes
- Place on the magnetic stand for 5 minutes
- Transfer supernatant

Second cDNA strand synthesis

- Add to a PCR tube:
  - 5µL NEB buffer (New England Biolabs)
  - 25µL of DNA
  - 3µL 10 µM Strand 2 cDNA Leish/Tryp primer
  - 14 µL water
- Incubate at 98°C for 5 minutes
- Slowly cool to room temperature (30 minutes) to room temperature by placing on the bench (primers will anneal as the reaction cools)
- Add:
  - 1 µL of Klenow fragment (New England Biolabs)
  - 2 µL of 20mM dNTP mix (5 mM each at neutral pH)
- Incubate at 37°C for 60 minutes
- Purify with AMPure XP beads (Agencourt)s as described above (add 90 µL of beads)
- Determine DNA concentration with Qubit

Addition of Adapters

- Add to a PCR tube:
  - 2.5 µL of DNA (5ng/µL)
  - 5µL of 1 µM forward primer
  - 5µL of 1 µM reverse primer
  - 12.5 µL of 2x HiFI Hotstart Ready Mix (KAPA)
- PCR 95°C – 5 min + (Denaturation 98°C – 20s + Annealing 55°C – 30s + 72°C -60sec)*18 cycles + Final extension 72°C – 5 minutes.
- Purify with AMPure XP beads (Agencourt) as described above (add 45 µL of beads)

Reverse primer for Leishmania:

5'-GTCTCGTGGGCTCGGAGATGTGTATAAGAGACAG<u>ATCAGTTTCTGTACTTTA</u> *

Forward primer:

*TCGTCGGCAGCGTCAGATGTGTATAAGAGACAG**

* Oligonucleotide sequences © 2016 Illumina, Inc. All rights reserved. Derivative works created by Illumina customers are authorized for use with Illumina instruments and products only. All other uses are strictly prohibited. The underlined part

Indexing with Nextera XT Index Kit

- Transfer 5µL of DNA to a new PCR tube
  - Add 5 µL of index 1 and 5 µL of index 2
  - Add 25 µL of 2x HiFi Hotstart Readry Mix (KAPA)
  - Add 10 µL of water and pipet up and down 10 times
- PCR 95°C for 3 minutes + 8 cycles of (Denaturation 98°C – 20s + Annealing 55°C – 30s + 72°C - 60sec) + 72°C for 5 minutes.
- Purify with AMPure XP beads (Agencourt) as described above (add 90 µL of beads)

Sequencing

- Use KAPA Library Quantification kit for Illumina systems to determine library concentration.
- Sequencing on Illumina platform. MiSeq, HiSeq, NextSeq, or more recent. Library loading concentration depends on the platform, we used 15 pM on both the MiSeq and HiSeq platform and achieved good clustering.

BLAST ® » blastn suite » RID-941W2WR001R

BLAST Results

Job title: Nucleotide Sequence (39 letters)

**RID**	941W2WR001R (Expires on 02-02 23:06 pm)
**Query ID**	lcl|Query_237925
**Description**	None
**Molecule type**	nucleic acid
**Query Length**	39
**Database Name**	nr
**Description**	Nucleotide collection (nt)
**Program**	BLASTN 2.6.1+

Graphic Summary

Distribution of the top 124 Blast Hits on 380 subject sequences

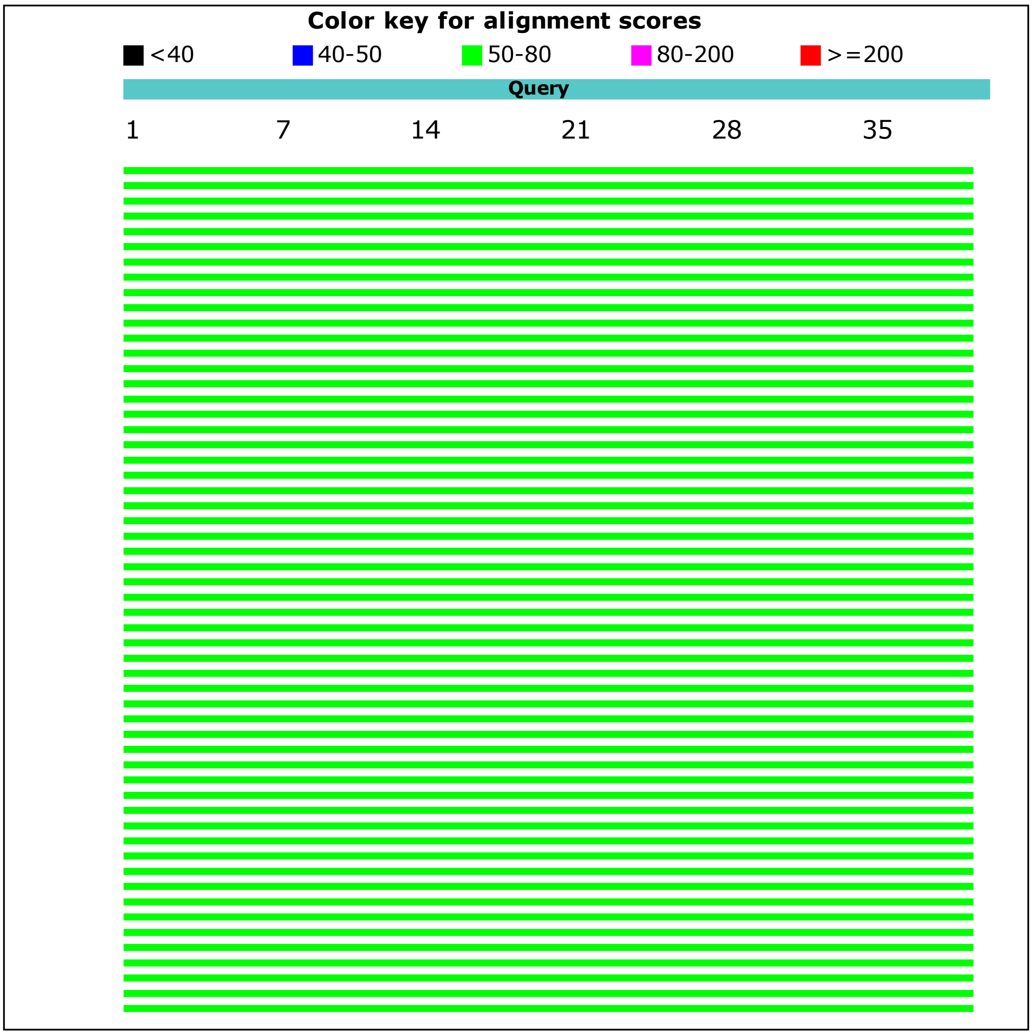

Descriptions

Sequences producing significant alignments:

Description	Max score	Total score	Query cover	E value	Ident	Accession
Uncultured trypanosome isolate 435AR-B-env trans-spliced leader sequence (SL) gene, complete sequence	73.1	73.1	100%	1e-10	100%	KR056279.1
Uncultured trypanosome isolate 434AR-B-env trans-spliced leader sequence (SL) gene, complete sequence	73.1	73.1	100%	1e-10	100%	KR056277.1
Uncultured trypanosome isolate 425LB-env trans-spliced leader sequence (SL) gene, complete sequence	73.1	73.1	100%	1e-10	100%	KR056271.1
Uncultured trypanosome isolate 405LB-env trans-spliced leader sequence (SL) gene, complete sequence	73.1	73.1	100%	1e-10	100%	KR056263.1
Uncultured trypanosome isolate 404LB-env trans-spliced leader sequence (SL) gene, complete sequence	73.1	73.1	100%	1e-10	100%	KR056262.1
Uncultured trypanosome isolate 346VL-B-env trans-spliced leader sequence (SL) gene, complete sequence	73.1	73.1	100%	1e-10	100%	KR056231.1
Uncultured trypanosome isolate 316AR-cult2 trans-spliced leader sequence (SL) gene, complete sequence	73.1	73.1	100%	1e-10	100%	KR056220.1
Uncultured trypanosome isolate 279OT-env trans-spliced leader sequence (SL) gene, complete sequence	73.1	73.1	100%	1e-10	100%	KR056218.1
Leptomonas pyrrhocoris isolate G58.02 trans-spliced leader (SL) gene, complete sequence	73.1	73.1	100%	1e-10	100%	KT012505.1
Leptomonas pyrrhocoris isolate SERG.02 trans-spliced leader (SL) gene, complete sequence	73.1	73.1	100%	1e-10	100%	KT012504.1
Leptomonas pyrrhocoris isolate SERG.01 trans-spliced leader (SL) gene, complete sequence	73.1	73.1	100%	1e-10	100%	KT012503.1
Leptomonas pyrrhocoris isolate K06.01 trans-spliced leader (SL) gene, complete sequence	73.1	73.1	100%	1e-10	100%	KT012502.1
Leptomonas pyrrhocoris isolate 121AL.01 trans-spliced leader (SL) gene, complete sequence	73.1	73.1	100%	1e-10	100%	KT012501.1
Leptomonas pyrrhocoris isolate 122AL.01 trans-spliced leader (SL) gene, complete sequence	73.1	73.1	100%	1e-10	100%	KT012500.1
Leptomonas pyrrhocoris isolate 324RV.02 trans-spliced leader (SL) gene, complete sequence	73.1	73.1	100%	1e-10	100%	KT012499.1
Leptomonas pyrrhocoris isolate 10VL.02 trans-spliced leader (SL) gene, complete sequence	73.1	73.1	100%	1e-10	100%	KT012498.1
Leptomonas pyrrhocoris isolate F165.02 trans-spliced leader (SL) gene, complete sequence	73.1	73.1	100%	1e-10	100%	KT012497.1
Leptomonas pyrrhocoris isolate F165.01 trans-spliced leader (SL) gene, complete sequence	73.1	73.1	100%	1e-10	100%	KT012496.1
Leptomonas pyrrhocoris isolate P59.02 trans-spliced leader (SL) gene, complete sequence	73.1	73.1	100%	1e-10	100%	KT012495.1
Leptomonas pyrrhocoris isolate P59.01 trans-spliced leader (SL) gene, complete sequence	73.1	73.1	100%	1e-10	100%	KT012494.1

Description	Max score	Total score	Query cover	E value	Ident	Accession
Leptomonas pyrrhocoris isolate F19.02 trans-spliced leader (SL) gene, complete sequence	73.1	73.1	100%	1e-10	100%	KT012493.1
Leptomonas pyrrhocoris isolate F19.01 trans-spliced leader (SL) gene, complete sequence	73.1	73.1	100%	1e-10	100%	KT012492.1
Leptomonas pyrrhocoris isolate CH278.02 trans-spliced leader (SL) gene, complete sequence	73.1	73.1	100%	1e-10	100%	KT012491.1
Leptomonas pyrrhocoris isolate CH278.01 trans-spliced leader (SL) gene, complete sequence	73.1	73.1	100%	1e-10	100%	KT012490.1
Leptomonas pyrrhocoris isolate 25EC.02 trans-spliced leader (SL) gene, complete sequence	73.1	73.1	100%	1e-10	100%	KT012489.1
Leptomonas pyrrhocoris isolate 25EC.01 trans-spliced leader (SL) gene, complete sequence	73.1	73.1	100%	1e-10	100%	KT012488.1
Leptomonas pyrrhocoris isolate 329MV.01 trans-spliced leader (SL) gene, complete sequence	73.1	73.1	100%	1e-10	100%	KT012487.1
Leptomonas pyrrhocoris isolate 14BT.02 trans-spliced leader (SL) gene, complete sequence	73.1	73.1	100%	1e-10	100%	KT012486.1
Leptomonas pyrrhocoris isolate 14BT.01 trans-spliced leader (SL) gene, complete sequence	73.1	73.1	100%	1e-10	100%	KT012485.1
Trypanosomatidae sp. isolate 280OT clone 2 trans-spliced leader (SL) gene, complete sequence	73.1	73.1	100%	1e-10	100%	KP717873.1
Trypanosomatidae sp. isolate 279OT clone 1 trans-spliced leader (SL) gene, complete sequence	73.1	140	100%	1e-10	100%	KP717871.1
Trypanosomatidae sp. isolate 262AT clone 1 trans-spliced leader (SL) gene, complete sequence	73.1	73.1	100%	1e-10	100%	KP717858.1
Trypanosomatidae sp. isolate 243VB clone 7 trans-spliced leader (SL) gene, complete sequence	73.1	73.1	100%	1e-10	100%	KP717838.1
Trypanosomatidae sp. isolate 240VB clone 4 trans-spliced leader (SL) gene, complete sequence	73.1	73.1	100%	1e-10	100%	KP717836.1
Trypanosomatidae sp. isolate 240VB clone 3 trans-spliced leader (SL) gene, complete sequence	73.1	73.1	100%	1e-10	100%	KP717835.1
Trypanosomatidae sp. isolate 237VB clone 4 trans-spliced leader (SL) gene, complete sequence	73.1	73.1	100%	1e-10	100%	KP717832.1
Trypanosomatidae sp. isolate 236VB clone 2B trans-spliced leader (SL) gene, complete sequence	73.1	73.1	100%	1e-10	100%	KP717828.1
Trypanosomatidae sp. isolate 219VB clone 4 trans-spliced leader (SL) gene, complete sequence	73.1	73.1	100%	1e-10	100%	KP717797.1
Trypanosomatidae sp. isolate 216BN clone 4 trans-spliced leader (SL) gene, complete sequence	73.1	73.1	100%	1e-10	100%	KP717794.1
Trypanosomatidae sp. isolate 192MD clone 1 trans-spliced leader (SL) gene, complete sequence	73.1	73.1	100%	1e-10	100%	KP717769.1
Trypanosomatidae sp. isolate 191MD clone 1 trans-spliced leader (SL) gene, complete sequence	73.1	73.1	100%	1e-10	100%	KP717768.1
Trypanosomatidae sp. isolate 190MD clone 1 trans-spliced leader (SL) gene, complete sequence	73.1	73.1	100%	1e-10	100%	KP717765.1

Description	Max score	Total score	Query cover	E value	Ident	Accession
Trypanosomatidae sp. isolate 189MD clone 9 trans-spliced leader (SL) gene, complete sequence	73.1	73.1	100%	1e-10	100%	KP717764.1
Leishmania peruviana genome assembly Leishmania peruviana PAB-4377_V1, chromosome : 0	73.1	1659	100%	1e-10	100%	LN609266.1
Trypanosomatidae sp. ECU-07 trans-spliced leader sequence SL var 1 gene, complete sequence	73.1	73.1	100%	1e-10	100%	KM242078.1
Crithidia brevicula isolate 101O clone 3 trans-spliced leader sequence (SL) gene, complete sequence	73.1	73.1	100%	1e-10	100%	KJ474937.1
Crithidia brevicula isolate 101O clone 2 trans-spliced leader sequence (SL) gene, complete sequence	73.1	73.1	100%	1e-10	100%	KJ474936.1
Crithidia brevicula isolate 101O clone 1 trans-spliced leader sequence (SL) gene, complete sequence	73.1	73.1	100%	1e-10	100%	KJ474935.1
Crithidia brevicula isolate Nbr clone 1 trans-spliced leader sequence (SL) gene, complete sequence	73.1	73.1	100%	1e-10	100%	KJ474931.1
Crithidia brevicula isolate KVI clone 2 trans-spliced leader sequence (SL) gene, complete sequence	73.1	73.1	100%	1e-10	100%	KJ474929.1
Crithidia brevicula isolate KVI clone 1 trans-spliced leader sequence (SL) gene, complete sequence	73.1	73.1	100%	1e-10	100%	KJ474928.1
Crithidia brevicula isolate CL8 clone 3 trans-spliced leader sequence (SL) gene, complete sequence	73.1	73.1	100%	1e-10	100%	KJ474927.1
Crithidia brevicula isolate CL8 clone 2 trans-spliced leader sequence (SL) gene, complete sequence	73.1	73.1	100%	1e-10	100%	KJ474926.1
Crithidia brevicula isolate CL8 clone 1 trans-spliced leader sequence (SL) gene, complete sequence	73.1	73.1	100%	1e-10	100%	KJ474925.1
Crithidia brevicula isolate F5 clone 2 trans-spliced leader sequence (SL) gene, complete sequence	73.1	73.1	100%	1e-10	100%	KJ474924.1
Crithidia brevicula isolate F8 clone 4 trans-spliced leader sequence (SL) gene, complete sequence	73.1	73.1	100%	1e-10	100%	KJ474922.1
Crithidia brevicula isolate F8 clone 3 trans-spliced leader sequence (SL) gene, complete sequence	73.1	73.1	100%	1e-10	100%	KJ474921.1
Crithidia brevicula isolate F8 clone 2 trans-spliced leader sequence (SL) gene, complete sequence	73.1	73.1	100%	1e-10	100%	KJ474920.1
Crithidia brevicula isolate F8 clone 1 trans-spliced leader sequence (SL) gene, complete sequence	73.1	73.1	100%	1e-10	100%	KJ474919.1
Crithidia brevicula isolate BM clone 3 trans-spliced leader sequence (SL) gene, complete sequence	73.1	73.1	100%	1e-10	100%	KJ474918.1
Crithidia brevicula isolate BM clone 2 trans-spliced leader sequence (SL) gene, complete sequence	73.1	73.1	100%	1e-10	100%	KJ474917.1
Crithidia brevicula isolate F2 clone 3 trans-spliced leader sequence (SL) gene, complete sequence	73.1	73.1	100%	1e-10	100%	KJ474915.1
Crithidia brevicula isolate F2 clone 2 trans-spliced leader sequence (SL) gene, complete sequence	73.1	73.1	100%	1e-10	100%	KJ474914.1
Crithidia brevicula isolate F2 clone 1 trans-spliced leader sequence (SL) gene, complete sequence	73.1	73.1	100%	1e-10	100%	KJ474913.1

Description	Max score	Total score	Query cover	E value	Ident	Accession
Crithidia brevicula isolate F7 clone 1 trans-spliced leader sequence (SL) gene, complete sequence	73.1	73.1	100%	1e-10	100%	KJ474910.1
Crithidia brevicula isolate Wg clone 3 trans-spliced leader sequence (SL) gene, complete sequence	73.1	73.1	100%	1e-10	100%	KJ474909.1
Crithidia brevicula isolate Wg clone 2 trans-spliced leader sequence (SL) gene, complete sequence	73.1	73.1	100%	1e-10	100%	KJ474908.1
Crithidia brevicula isolate Wg clone 1 trans-spliced leader sequence (SL) gene, complete sequence	73.1	73.1	100%	1e-10	100%	KJ474907.1
Crithidia brevicula isolate ZK clone 3 trans-spliced leader sequence (SL) gene, complete sequence	73.1	73.1	100%	1e-10	100%	KJ474906.1
Crithidia brevicula isolate ZK clone 1 trans-spliced leader sequence (SL) gene, complete sequence	73.1	73.1	100%	1e-10	100%	KJ474904.1
Crithidia brevicula isolate F6 clone 3 trans-spliced leader sequence (SL) gene, complete sequence	73.1	73.1	100%	1e-10	100%	KJ474903.1
Crithidia brevicula isolate F6 clone 2 trans-spliced leader sequence (SL) gene, complete sequence	73.1	73.1	100%	1e-10	100%	KJ474902.1
Crithidia brevicula isolate F6 clone 1 trans-spliced leader sequence (SL) gene, complete sequence	73.1	73.1	100%	1e-10	100%	KJ474901.1
Crithidia otongatchiensis isolate G15 trans-spliced leader sequence SL gene, complete sequence	73.1	73.1	100%	1e-10	100%	KF482066.1
Leptomonas moramango isolate MMO-09 trans-spliced leader sequence SL gene, complete sequence	73.1	73.1	100%	1e-10	100%	KF482064.1
Crithidia otongatchiensis isolate ECU-08 trans-spliced leader sequence SL gene, complete sequence	73.1	73.1	100%	1e-10	100%	KF482063.1
Blechomonas campbelli isolate B08-CTE2/A trans-spliced leader sequence SL gene, complete sequence	73.1	73.1	100%	1e-10	100%	KF054159.1
Trypanosomatidae sp. G40 trans-spliced leader sequence SL gene, complete sequence	73.1	73.1	100%	1e-10	100%	JQ890157.1
Trypanosomatidae sp. G37 trans-spliced leader sequence SL gene, complete sequence	73.1	73.1	100%	1e-10	100%	JQ890151.1
Trypanosomatidae sp. G32 trans-spliced leader sequence SL gene, complete sequence	73.1	73.1	100%	1e-10	100%	JQ890145.1
Trypanosomatidae sp. G22 trans-spliced leader sequence SL gene, complete sequence	73.1	73.1	100%	1e-10	100%	JQ890131.1
Trypanosomatidae sp. G15ax trans-spliced leader sequence SL gene, complete sequence	73.1	73.1	100%	1e-10	100%	JQ890123.1
Trypanosomatidae sp. E07 trans-spliced leader sequence SL gene, complete sequence	73.1	73.1	100%	1e-10	100%	JQ890111.1
Crithidia brachyflagelli isolate 343VL trans-spliced leader sequence (SL) gene, complete sequence	73.1	73.1	100%	1e-10	100%	JF734909.1
Crithidia brachyflagelli isolate 343VL-2 trans-spliced leader sequence (SL) gene, complete sequence	73.1	73.1	100%	1e-10	100%	JF734908.1
Crithidia brachyflagelli isolate 342VL trans-spliced leader sequence (SL) gene, complete sequence	73.1	73.1	100%	1e-10	100%	JF734906.1

Description	Max score	Total score	Query cover	E value	Ident	Accession
Leptomonas spiculata isolate 331MV trans-spliced leader sequence (SL) gene, complete sequence	73.1	73.1	100%	1e-10	100%	JF734889.1
Leptomonas pyrrhocoris isolate G7 trans-spliced leader sequence (SL) gene, complete sequence	73.1	73.1	100%	1e-10	100%	JF950610.1
Leptomonas pyrrhocoris isolate G59 trans-spliced leader sequence (SL) gene, complete sequence	73.1	73.1	100%	1e-10	100%	JF950609.1
Leptomonas pyrrhocoris isolate G58 trans-spliced leader sequence (SL) gene, complete sequence	73.1	73.1	100%	1e-10	100%	JF950608.1
Leptomonas pyrrhocoris isolate KYPR10 trans-spliced leader sequence (SL) gene, complete sequence	73.1	73.1	100%	1e-10	100%	JF950606.1
Leptomonas pyrrhocoris isolate P59 trans-spliced leader sequence (SL) gene, complete sequence	73.1	73.1	100%	1e-10	100%	JF950605.1
Leptomonas pyrrhocoris isolate F19 trans-spliced leader sequence (SL) gene, complete sequence	73.1	73.1	100%	1e-10	100%	JF950603.1
Leptomonas pyrrhocoris isolate SK03 trans-spliced leader sequence (SL) gene, complete sequence	73.1	73.1	100%	1e-10	100%	JF950602.1
Leptomonas pyrrhocoris isolate G07 trans-spliced leader sequence (SL) gene, complete sequence	73.1	73.1	100%	1e-10	100%	JF950597.1
Leptomonas pyrrhocoris isolate H10 trans-spliced leader sequence (SL) gene, complete sequence	73.1	73.1	100%	1e-10	100%	JF950595.1
Leptomonas pyrrhocoris isolate 122AL trans-spliced leader sequence (SL) gene, complete sequence	73.1	73.1	100%	1e-10	100%	JF937087.1
Leptomonas pyrrhocoris isolate 80MV trans-spliced leader sequence (SL) gene, complete sequence	73.1	73.1	100%	1e-10	100%	JF937086.1
Leptomonas pyrrhocoris isolate 28EC-C trans-spliced leader sequence (SL) gene, complete sequence	73.1	73.1	100%	1e-10	100%	JF937084.1
Leptomonas pyrrhocoris isolate 28EC-B trans-spliced leader sequence (SL) gene, complete sequence	73.1	73.1	100%	1e-10	100%	JF937083.1
Leptomonas pyrrhocoris isolate 28EC-A trans-spliced leader sequence (SL) gene, complete sequence	73.1	73.1	100%	1e-10	100%	JF937082.1
Leptomonas pyrrhocoris isolate 25EC trans-spliced leader sequence (SL) gene, complete sequence	73.1	73.1	100%	1e-10	100%	JF937081.1
Leptomonas pyrrhocoris isolate 14BT-C trans-spliced leader sequence (SL) gene, complete sequence	73.1	73.1	100%	1e-10	100%	JF937080.1
Leptomonas pyrrhocoris isolate 14BT-B trans-spliced leader sequence (SL) gene, complete sequence	73.1	73.1	100%	1e-10	100%	JF937079.1
Leptomonas pyrrhocoris isolate 14BT-A trans-spliced leader sequence (SL) gene, complete sequence	73.1	73.1	100%	1e-10	100%	JF937078.1
Leptomonas pyrrhocoris isolate 12VL trans-spliced leader sequence (SL) gene, complete sequence	73.1	73.1	100%	1e-10	100%	JF937077.1
Leptomonas pyrrhocoris isolate 10VL trans-spliced leader sequence (SL) gene, complete sequence	73.1	73.1	100%	1e-10	100%	JF937076.1
Leptomonas pyrrhocoris strain 124AL trans-spliced leader sequence (SL) gene, complete sequence	73.1	73.1	100%	1e-10	100%	JN036650.1

Description	Max score	Total score	Query cover	E value	Ident	Accession
Leptomonas scantii isolate F221 trans-spliced leader sequence (SL) gene, complete sequence	73.1	73.1	100%	1e-10	100%	JN009104.1
Leptomonas scantii isolate KYPR12 trans-spliced leader sequence (SL) gene, complete sequence	73.1	73.1	100%	1e-10	100%	JF950594.1
Leptomonas scantii isolate KYPR01 trans-spliced leader sequence (SL) gene, complete sequence	73.1	73.1	100%	1e-10	100%	JF950593.1
Leptomonas scantii isolate Tun trans-spliced leader sequence (SL) gene, complete sequence	73.1	73.1	100%	1e-10	100%	JF950592.1
Leptomonas pyrrhocoris isolate 326RV trans-spliced leader sequence (SL) gene, complete sequence	73.1	73.1	100%	1e-10	100%	JF937073.1
Leishmania donovani BPK282A1 complete genome, chromosome 2	73.1	146	100%	1e-10	100%	FR799589.2
Trypanosomatidae sp. LW-2010E1 clone 21_sub trans-spliced leader gene, partial sequence	73.1	73.1	100%	1e-10	100%	HQ285325.1
Leishmania mexicana MHOM/GT/2001/U1103 complete genome, chromosome 2	73.1	292	100%	1e-10	100%	FR799555.1
Leishmania major strain Friedlin complete genome, chromosome 2	73.1	4534	100%	1e-10	100%	FR796398.1
Leishmania infantum JPCM5 genome chromosome 2	73.1	658	100%	1e-10	100%	FR796434.1
Leptomonas pyrrhocoris clone 278Jl2_1 trans-spliced leader sequence SL gene, complete sequence	73.1	73.1	100%	1e-10	100%	GU063786.1
Leishmania amazonensis clone A'669 GTP binding protein-like mRNA, partial sequence	73.1	73.1	100%	1e-10	100%	EU429419.1
Leptomonas peterhoffi isolate 3 trans-spliced leader sequence SL (trans-spliced leader sequence SL) gene, complete sequence	73.1	73.1	100%	1e-10	100%	EU088285.1
Leptomonas peterhoffi isolate 2 trans-spliced leader sequence SL (trans-spliced leader sequence SL) gene, complete sequence	73.1	73.1	100%	1e-10	100%	EU088284.1
Leptomonas peterhoffi isolate 1 trans-spliced leader sequence SL (trans-spliced leader sequence SL) gene, complete sequence	73.1	73.1	100%	1e-10	100%	EU088283.1
Wallaceina inconstans isolate 3 trans-spliced leader sequence SL (trans-spliced leader sequence SL) gene, complete sequence	73.1	73.1	100%	1e-10	100%	EU088282.1
Wallaceina inconstans isolate 2 trans-spliced leader sequence SL (trans-spliced leader sequence SL) gene, complete sequence	73.1	73.1	100%	1e-10	100%	EU088281.1
Wallaceina inconstans isolate 1 trans-spliced leader sequence SL (trans-spliced leader sequence SL) gene, complete sequence	73.1	73.1	100%	1e-10	100%	EU088280.1
Wallaceina brevicula isolate 3 trans-spliced leader sequence SL (trans-spliced leader sequence SL) gene, complete sequence	73.1	73.1	100%	1e-10	100%	EU088279.1
Wallaceina brevicula isolate 2 trans-spliced leader sequence SL (trans-spliced leader sequence SL) gene, complete sequence	73.1	73.1	100%	1e-10	100%	EU088278.1
Wallaceina brevicula isolate 1 trans-spliced leader sequence SL (trans-spliced leader sequence SL) gene, complete sequence	73.1	73.1	100%	1e-10	100%	EU088277.1

Description	Max score	Total score	Query cover	E value	Ident	Accession
Crithidia permixta isolate 2 trans-spliced leader sequence SL (trans-spliced leader sequence SL) gene, complete sequence	73.1	73.1	100%	1e-10	100%	EU088276.1
Crithidia permixta isolate 128SI trans-spliced leader sequence SL (trans-spliced leader sequence SL) gene, complete sequence	73.1	73.1	100%	1e-10	100%	EU088275.1
Crithidia insperata isolate 119YS trans-spliced leader sequence SL (trans-spliced leader sequence SL) gene, complete sequence	73.1	73.1	100%	1e-10	100%	EU088272.1
Leptomonas cf. podlipaevi isolate 59LI trans-spliced leader sequence SL (trans-spliced leader sequence SL) gene, complete sequence	73.1	73.1	100%	1e-10	100%	EU088271.1
Leptomonas neopamerae isolate 73BR clone 1 trans-spliced leader sequence SL genomic sequence	73.1	73.1	100%	1e-10	100%	EF152332.1
Crithidia deanei clone 28 trans-spliced leader sequence SL	73.1	73.1	100%	1e-10	100%	EF546791.1
Leptomonas tarcoles trans-spliced leader sequence SL var 2	73.1	73.1	100%	1e-10	100%	EF546789.1
Leptomonas sp. acus. isolate 132SI trans-spliced leader sequence SL complete sequence	73.1	73.1	100%	1e-10	100%	EF152331.1
Trypanosomatidae sp. 80AR-D trans-spliced leader sequence gene, complete sequence	73.1	73.1	100%	1e-10	100%	DQ864333.1
Leptomonas pyrrhocoris isolate 80MV-E trans-spliced leader sequence gene, complete sequence	73.1	73.1	100%	1e-10	100%	DQ864332.1
Trypanosomatidae sp. 73BR-A trans-spliced leader sequence gene, complete sequence	73.1	73.1	100%	1e-10	100%	DQ864326.1
Trypanosomatidae sp. 71CN-A trans-spliced leader sequence gene, complete sequence	73.1	73.1	100%	1e-10	100%	DQ864323.1
Leptomonas barvae isolate 21EC-B trans-spliced leader sequence gene, complete sequence	73.1	73.1	100%	1e-10	100%	DQ864312.1
Leptomonas barvae isolate 19EC trans-spliced leader sequence gene, complete sequence	73.1	73.1	100%	1e-10	100%	DQ864311.1
Leptomonas pyrrhocoris isolate 14BT-B trans-spliced leader sequence gene, complete sequence	73.1	73.1	100%	1e-10	100%	DQ864309.1
Leptomonas pyrrhocoris isolate 10VL-C trans-spliced leader sequence gene, complete sequence	73.1	73.1	100%	1e-10	100%	DQ864308.1
Trypanosomatidae sp. 110SI trans-spliced leader sequence gene, complete sequence	73.1	73.1	100%	1e-10	100%	DQ864306.1
Trypanosomatidae sp. 104SI trans-spliced leader sequence gene, complete sequence	73.1	73.1	100%	1e-10	100%	DQ864304.1
Trypanosomatidae sp. 98SI trans-spliced leader sequence gene, complete sequence	73.1	73.1	100%	1e-10	100%	DQ864302.1
Trypanosomatidae sp. 90JS trans-spliced leader sequence gene, complete sequence	73.1	146	100%	1e-10	100%	DQ864296.1
Trypanosomatidae sp. 82AL-B trans-spliced leader sequence gene, complete sequence	73.1	73.1	100%	1e-10	100%	DQ864289.1
Trypanosomatidae sp. 82AL-A trans-spliced leader sequence gene, complete sequence	73.1	146	100%	1e-10	100%	DQ864288.1

Description	Max score	Total score	Query cover	E value	Ident	Accession
Trypanosomatidae sp. 136YS trans-spliced leader sequence gene, complete sequence	73.1	219	100%	1e-10	100%	DQ864274.1
Leptomonas costaricensis strain 15EC copy 1 trans-spliced leader sequence SL, complete sequence	73.1	73.1	100%	1e-10	100%	DQ977641.1
Trypanosomatidae sp. 50CR-A trans-spliced leader sequence, complete sequence	73.1	73.1	100%	1e-10	100%	DQ860230.1
Trypanosomatidae sp. 35EC-B trans-spliced leader sequence, complete sequence	73.1	73.1	100%	1e-10	100%	DQ860222.1
Leptomonas pyrrhocoris isolate 25EC-E trans-spliced leader sequence gene, complete sequence	73.1	73.1	100%	1e-10	100%	DQ860218.1
Leishmania donovani S-adenosyl-L-methionine-C-24-delta-sterol-methyltransferase A (SCMT1) mRNA, complete cds	73.1	73.1	100%	1e-10	100%	AY488058.1
Leishmania amazonensis meta 2 protein (meta2) mRNA, complete cds	73.1	73.1	100%	1e-10	100%	AY061813.2
Leptomonas podlipaevi subclone UCR5-26 trans-spliced leader sequence SL gene, complete sequence	73.1	73.1	100%	1e-10	100%	DQ140174.1
Leishmania donovani cyclin mRNA, complete cds	73.1	73.1	100%	1e-10	100%	AF531425.2
Leishmania tarentolae isolate LEM 125 locus MINA trans-spliced leader sequence SL gene, complete sequence	73.1	73.1	100%	1e-10	100%	AY100201.1
Leishmania tarentolae isolate LEM 125 locus MINB trans-spliced leader sequence SL gene, complete sequence	73.1	73.1	100%	1e-10	100%	AY100198.1
Leishmania adleri isolate LV 30 trans-spliced leader sequence SL gene, complete sequence	73.1	73.1	100%	1e-10	100%	AY100194.1
Leishmania hoogstraali isolate NG26 locus MINA trans-spliced leader sequence SL gene, complete sequence	73.1	73.1	100%	1e-10	100%	AY100197.1
Leishmania gymnodactyli isolate LV 247 locus MINB trans-spliced leader sequence SL gene, complete sequence	73.1	73.1	100%	1e-10	100%	AY100196.1
Leishmania gymnodactyli isolate LV 247 locus MINA trans-spliced leader sequence SL gene, complete sequence	73.1	73.1	100%	1e-10	100%	AY100195.1
Leptomonas sp. Cfm spliced leader RNA gene, partial sequence	73.1	73.1	100%	1e-10	100%	AY547465.1
Leptomonas sp. P spliced leader RNA gene, complete sequence	73.1	73.1	100%	1e-10	100%	AY547464.1
Leptomonas sp. C4 spliced leader RNA gene, complete sequence	73.1	73.1	100%	1e-10	100%	AY547463.1
Blastocrithidia gerricola spliced leader RNA gene, complete sequence	73.1	73.1	100%	1e-10	100%	AY547459.1
Leishmania donovani isoleucine tRNA synthetase mRNA, 5' UTR and partial cds	73.1	73.1	100%	1e-10	100%	AF464774.1
Leishmania donovani nonspecific nucleoside hydrolase mRNA, complete cds	73.1	73.1	100%	1e-10	100%	AY033633.1
Leishmania infantum mRNA for regulatory subunit of the HslVU complex (hslU gene), putative short splice variant	73.1	73.1	100%	1e-10	100%	AJ428522.1
Leishmania infantum mRNA for regulatory subunit of the HslVU complex (hslU gene), putative long splice variant	73.1	73.1	100%	1e-10	100%	AJ428521.1
Leishmania infantum mRNA for HslVU complex proteolytic subunit (hslV gene)	73.1	73.1	100%	1e-10	100%	AJ298867.1

Description	Max score	Total score	Query cover	E value	Ident	Accession
Leishmania donovani chaperonin TCP20 (TCP20) mRNA, complete cds	73.1	73.1	100%	1e-10	100%	AF322112.1
Leishmania infantum mRNA for proteasome alpha 1 subunit	73.1	73.1	100%	1e-10	100%	Y13060.1
Leishmania infantum mRNA for proteasome alpha-2 subunit	73.1	73.1	100%	1e-10	100%	Y13059.1
Leishmania infantum mRNA for eukaryotic initiation factor 5a (eIF5A gene)	73.1	73.1	100%	1e-10	100%	AJ278539.1
Leishmania infantum mRNA for ribosomal protein S33 (s33 gene)	73.1	73.1	100%	1e-10	100%	AJ278540.2
Crithidia fasciculata retrotransposon CRE 1 unknown gene	73.1	127	100%	1e-10	100%	M33009.2
Leishmania tarentolae histone H4 mRNA, complete cds	73.1	73.1	100%	1e-10	100%	AF175386.1
L.major mRNA for beta-tubulin (2190bp)	73.1	73.1	100%	1e-10	100%	X93566.1
Leishmania donovani isolate MHOM/ET/67/HU3 miniexon gene array	73.1	725	100%	1e-10	100%	AY557374.1
Leishmania infantum mRNA for ribosomal protein L15	73.1	73.1	100%	1e-10	100%	AJ276107.1
Leishmania infantum mRNA for putative ribosomal protein L10 (LiL10 gene)	73.1	73.1	100%	1e-10	100%	AJ278542.1
Leishmania infantum mRNA for ribosomal protein S25 (s25 gene)	73.1	73.1	100%	1e-10	100%	AJ278541.1
Leishmania infantum mRNA for hypothetical protein (26 kDa), (ORF1)	73.1	73.1	100%	1e-10	100%	AJ278538.1
Leishmania infantum mRNA for nascent polypeptide associated complex homologue, alpha chain	73.1	73.1	100%	1e-10	100%	AJ404610.1
Leishmania amazonensis 40S ribosomal protein S24e (S24e) mRNA, complete cds	73.1	73.1	100%	1e-10	100%	AF252289.1
Leishmania donovani 60S ribosomal protein L32 (RPL32) mRNA, complete cds	73.1	73.1	100%	1e-10	100%	AF241825.1
Leishmania donovani pyrroline-5-carboxylate reductase (P5CR) mRNA, complete cds	73.1	73.1	100%	1e-10	100%	AF241824.1
Leishmania amazonensis 60S ribosomal protein L44 (RPL44) mRNA, complete cds	73.1	73.1	100%	1e-10	100%	AF148853.2
Leishmania donovani infantum mini-exon copy C, complete sequence	73.1	73.1	100%	1e-10	100%	AF097655.1
Leishmania donovani infantum mini-exon copy B, complete sequence	73.1	73.1	100%	1e-10	100%	AF097654.1
Leishmania donovani infantum mini-exon copy A, complete sequence	73.1	73.1	100%	1e-10	100%	AF097653.1
Leishmania tarentolae DNA for mini-exon, 349 bp	73.1	73.1	100%	1e-10	100%	X97622.1
Leishmania tarentolae DNA for mini-exon, 296 bp	73.1	73.1	100%	1e-10	100%	X97620.1
Leishmania tarentolae DNA for mini-exon, 350 bp	73.1	73.1	100%	1e-10	100%	X97618.1
Leishmania tarentolae DNA for mini-exon, 363 bp	73.1	73.1	100%	1e-10	100%	X97617.1
Leishmania major activated protein kinase C receptor homolog LACK mRNA, complete cds	73.1	73.1	100%	1e-10	100%	U27568.1
Leishmania infantum ribosomal protein L37 (rpl37alpha) mRNA, complete cds	73.1	73.1	100%	1e-10	100%	U35460.1
Crithidia fasciculata retrotransposon CRE2 in mini-exon gene, putative reverse transcriptase gene, complete cds	73.1	127	100%	1e-10	100%	U19151.1
L.tarentolae mini-exon for medRNA	73.1	73.1	100%	1e-10	100%	X73121.1

Description	Max score	Total score	Query cover	E value	Ident	Accession
C.fasciculata mini-exon gene repeat	73.1	73.1	100%	1e-10	100%	J03470.1
L.major DNA for spliced leader sequence of kinetoplast mRNAs 5' term	73.1	73.1	100%	1e-10	100%	X58600.1
Leptomonas seymouri mini-exon donor RNA (medRNA)	73.1	73.1	100%	1e-10	100%	X07487.1
L.mexicana spliced leader RNA gene	73.1	133	100%	1e-10	100%	X64317.1
L.donovani mini exon derived RNA	73.1	73.1	100%	1e-10	100%	X62143.1
Crithidia fasciculata cf-2 Hind III mini-exon repeat	73.1	73.1	100%	1e-10	100%	X05258.1
Trypanosomatidae sp. AK-2016a strain E262-AT clone 2 trans-spliced leader sequence gene, complete sequence	69.4	69.4	100%	2e-09	97%	KT944299.1
Trypanosomatidae sp. AK-2016a strain E262-AT clone 1 trans-spliced leader sequence gene, complete sequence	69.4	69.4	100%	2e-09	97%	KT944298.1
Uncultured trypanosome isolate 438AR-env trans-spliced leader sequence (SL) gene, complete sequence	67.6	67.6	100%	6e-09	97%	KR056281.1
Uncultured trypanosome isolate 435AR-A-env trans-spliced leader sequence (SL) gene, complete sequence	67.6	67.6	100%	6e-09	97%	KR056278.1
Uncultured trypanosome isolate 423LB-env trans-spliced leader sequence (SL) gene, complete sequence	67.6	67.6	100%	6e-09	97%	KR056269.1
Uncultured trypanosome isolate 403LB-env trans-spliced leader sequence (SL) gene, complete sequence	67.6	67.6	100%	6e-09	97%	KR056261.1
Uncultured trypanosome isolate 400LB-env trans-spliced leader sequence (SL) gene, complete sequence	67.6	67.6	100%	6e-09	97%	KR056260.1
Uncultured trypanosome isolate 379VL-env trans-spliced leader sequence (SL) gene, complete sequence	67.6	67.6	100%	6e-09	97%	KR056249.1
Uncultured trypanosome isolate 335VL-A-env trans-spliced leader sequence (SL) gene, complete sequence	67.6	67.6	100%	6e-09	97%	KR056226.1
Trypanosomatidae sp. isolate 303OT clone 1 trans-spliced leader (SL) gene, complete sequence	67.6	67.6	100%	6e-09	97%	KP717889.1
Trypanosomatidae sp. isolate 285OT clone 1 trans-spliced leader (SL) gene, complete sequence	67.6	67.6	100%	6e-09	97%	KP717882.1
Trypanosomatidae sp. isolate 237VB clone 9 trans-spliced leader (SL) gene, complete sequence	67.6	67.6	100%	6e-09	97%	KP717833.1
Trypanosomatidae sp. isolate 236VB clone 2A trans-spliced leader (SL) gene, complete sequence	67.6	67.6	100%	6e-09	97%	KP717827.1
Trypanosomatidae sp. isolate 231VB clone 6 trans-spliced leader (SL) gene, complete sequence	67.6	67.6	100%	6e-09	97%	KP717814.1
Trypanosomatidae sp. isolate 224VB clone 2 trans-spliced leader (SL) gene, complete sequence	67.6	67.6	100%	6e-09	97%	KP717803.1
Trypanosomatidae sp. isolate 219VB clone 9 trans-spliced leader (SL) gene, complete sequence	67.6	67.6	100%	6e-09	97%	KP717799.1
Trypanosomatidae sp. isolate 215BN clone 1 trans-spliced leader (SL) gene, complete sequence	67.6	67.6	100%	6e-09	97%	KP717791.1
Trypanosomatidae sp. isolate 209AL clone 1 trans-spliced leader (SL) gene, complete sequence	67.6	67.6	100%	6e-09	97%	KP717779.1

Description	Max score	Total score	Query cover	E value	Ident	Accession
Trypanosomatidae sp. isolate 193MD clone 9 trans-spliced leader (SL) gene, complete sequence	67.6	67.6	100%	6e-09	97%	KP717770.1
Leishmania peruviana genome assembly Leishmania peruviana LEM-1537_V1, chromosome : 17	67.6	67.6	92%	6e-09	100%	LN609218.1
Crithidia brevicula isolate 101N clone 2 trans-spliced leader sequence (SL) gene, complete sequence	67.6	67.6	100%	6e-09	97%	KJ474934.1
Crithidia brevicula isolate 101N clone 1 trans-spliced leader sequence (SL) gene, complete sequence	67.6	67.6	100%	6e-09	97%	KJ474933.1
Crithidia brevicula isolate KVI clone 3 trans-spliced leader sequence (SL) gene, complete sequence	67.6	67.6	100%	6e-09	97%	KJ474930.1
Crithidia brevicula isolate F5 clone 1 trans-spliced leader sequence (SL) gene, complete sequence	67.6	67.6	100%	6e-09	97%	KJ474923.1
Crithidia brevicula isolate F7 clone 3 trans-spliced leader sequence (SL) gene, complete sequence	67.6	67.6	100%	6e-09	97%	KJ474912.1
Crithidia brevicula isolate F7 clone 2 trans-spliced leader sequence (SL) gene, complete sequence	67.6	67.6	100%	6e-09	97%	KJ474911.1
Leptomonas tenua isolate B08-466 trans-spliced leader sequence SL gene, complete sequence	67.6	67.6	100%	6e-09	97%	KF054152.1
Leptomonas tenua isolate B08-481/A trans-spliced leader sequence SL gene, complete sequence	67.6	67.6	100%	6e-09	97%	KF054151.1
Crithidia brachyflagelli isolate 343VL-1 trans-spliced leader sequence (SL) gene, complete sequence	67.6	67.6	100%	6e-09	97%	JF734907.1
Crithidia brachyflagelli isolate 342VL trans-spliced leader sequence (SL) gene, complete sequence	67.6	67.6	100%	6e-09	97%	JF734905.1
Leptomonas tenua isolate 341VL trans-spliced leader sequence (SL) gene, complete sequence	67.6	67.6	100%	6e-09	97%	JF734904.1
Leptomonas tenua isolate 341VL trans-spliced leader sequence (SL) gene, complete sequence	67.6	67.6	100%	6e-09	97%	JF734903.1
Crithidia brachyflagelli isolate 340VL trans-spliced leader sequence (SL) gene, complete sequence	67.6	67.6	100%	6e-09	97%	JF734902.1
Crithidia brachyflagelli isolate 340VL trans-spliced leader sequence (SL) gene, complete sequence	67.6	67.6	100%	6e-09	97%	JF734901.1
Leptomonas tenua isolate 339VL trans-spliced leader sequence (SL) gene, complete sequence	67.6	67.6	100%	6e-09	97%	JF734900.1
Trypanosomatidae sp. 338VL isolate 338VL-B trans-spliced leader sequence (SL) gene, complete sequence	67.6	67.6	100%	6e-09	97%	JF734899.1
Leptomonas tenua isolate 337VL trans-spliced leader sequence (SL) gene, complete sequence	67.6	67.6	100%	6e-09	97%	JF734897.1
Leptomonas tenua isolate 337VL-2 trans-spliced leader sequence (SL) gene, complete sequence	67.6	67.6	100%	6e-09	97%	JF734896.1
Leptomonas tenua isolate 337VL-1 trans-spliced leader sequence (SL) gene, complete sequence	67.6	67.6	100%	6e-09	97%	JF734895.1
Leptomonas spiculata isolate 333MV trans-spliced leader sequence (SL) gene, complete sequence	67.6	67.6	100%	6e-09	97%	JF734893.1

Description	Max score	Total score	Query cover	E value	Ident	Accession
Leptomonas spiculata isolate 331MV trans-spliced leader sequence (SL) gene, complete sequence	67.6	67.6	100%	6e-09	97%	JF734890.1
Crithidia confusa isolate 320AR trans-spliced leader sequence (SL) gene, complete sequence	67.6	67.6	100%	6e-09	97%	JF734887.1
Crithidia insperata isolate 316AR trans-spliced leader sequence (SL) gene, complete sequence	67.6	67.6	100%	6e-09	97%	JF734885.1
Leptomonas pyrrhocoris isolate 57VL trans-spliced leader sequence (SL) gene, complete sequence	67.6	67.6	100%	6e-09	97%	JF937085.1
Leptomonas pyrrhocoris isolate 329MV-B trans-spliced leader sequence (SL) gene, complete sequence	67.6	67.6	100%	6e-09	97%	JF937075.1
Leptomonas pyrrhocoris isolate 329MV trans-spliced leader sequence (SL) gene, complete sequence	67.6	67.6	100%	6e-09	97%	JF937074.1
Leptomonas pyrrhocoris isolate 326RV trans-spliced leader sequence (SL) gene, complete sequence	67.6	67.6	100%	6e-09	97%	JF937072.1
Leptomonas pyrrhocoris isolate 325RV trans-spliced leader sequence (SL) gene, complete sequence	67.6	67.6	100%	6e-09	97%	JF937071.1
Leptomonas pyrrhocoris isolate 325RV trans-spliced leader sequence (SL) gene, complete sequence	67.6	67.6	100%	6e-09	97%	JF937070.1
Leptomonas pyrrhocoris isolate 324RV trans-spliced leader sequence (SL) gene, complete sequence	67.6	67.6	100%	6e-09	97%	JF937068.1
Trypanosomatidae sp. Ch10 clone 391JI1 trans-spliced leader sequence SL, complete sequence	67.6	67.6	100%	6e-09	97%	GU063798.1
Leptomonas pyrrhocoris clone 282JI2 trans-spliced leader sequence SL gene, complete sequence	67.6	67.6	100%	6e-09	97%	GU063787.1
Crithidia abscondita isolate 127AL trans-spliced leader sequence SL (trans-spliced leader sequence SL) gene, complete sequence	67.6	67.6	100%	6e-09	97%	EU088274.1
Leptomonas jaderae isolate 34EC trans-spliced leader sequence SL (trans-spliced leader sequence SL) gene, complete sequence	67.6	67.6	100%	6e-09	97%	EU088270.1
Crithidia deanei clone 29 trans-spliced leader sequence SL	67.6	67.6	100%	6e-09	97%	EF546790.1
Leptomonas tarcoles trans-spliced leader sequence SL var 1 gene, complete sequence	67.6	67.6	100%	6e-09	97%	EF546788.1
Leptomonas bifurcata isolate 53CR isolate 53CR trans-spliced leader sequence SL complete sequence	67.6	67.6	100%	6e-09	97%	EF152330.1
Leptomonas barvae isolate 22EC-A trans-spliced leader sequence gene, complete sequence	67.6	67.6	100%	6e-09	97%	DQ864313.1
Trypanosomatidae sp. 140YS trans-spliced leader sequence gene, complete sequence	67.6	67.6	100%	6e-09	97%	DQ864277.1
Trypanosomatidae sp. 137YS trans-spliced leader sequence gene, complete sequence	67.6	67.6	100%	6e-09	97%	DQ864275.1
Trypanosomatidae sp. 132SI-A trans-spliced leader sequence gene, complete sequence	67.6	67.6	100%	6e-09	97%	DQ864270.1
Trypanosomatidae sp. 120YS-A trans-spliced leader sequence gene, complete sequence	67.6	67.6	100%	6e-09	97%	DQ864264.1

Description	Max score	Total score	Query cover	E value	Ident	Accession
Leptomonas costaricensis strain 15EC copy 2 trans-spliced leader sequence SL, complete sequence	67.6	67.6	100%	6e-09	97%	DQ977642.1
Trypanosomatidae sp. 55VL-B trans-spliced leader sequence, complete sequence	67.6	67.6	100%	6e-09	97%	DQ860232.1
Trypanosomatidae sp. 47VL-B trans-spliced leader sequence, complete sequence	67.6	67.6	100%	6e-09	97%	DQ860227.1
Leptomonas podlipaevi subclone UCR5-28 trans-spliced leader sequence SL gene, complete sequence	67.6	67.6	100%	6e-09	97%	DQ140175.1
Leptomonas podlipaevi subclone UCR4-21 trans-spliced leader sequence SL gene, complete sequence	67.6	67.6	100%	6e-09	97%	DQ140173.1
Leptomonas podlipaevi subclone UCR4-20 trans-spliced leader sequence SL gene, complete sequence	67.6	67.6	100%	6e-09	97%	DQ140172.1
Leptomonas podlipaevi subclone UCR4-19 trans-spliced leader sequence SL gene, complete sequence	67.6	67.6	100%	6e-09	97%	DQ140171.1
Leptomonas sp. F2 trans-spliced leader sequence SL gene, complete sequence	67.6	67.6	100%	6e-09	97%	DQ140170.1
Leishmania tropica pgfs mRNA forProstaglandin F2-alpha synthase, complete cds	67.6	67.6	92%	6e-09	100%	AB079546.1
Leishmania donovani pgfs mRNA forProstaglandin F2-alpha synthase, complete cds	67.6	67.6	92%	6e-09	100%	AB079545.1
Crithidia brevicula isolate ZK clone 2 trans-spliced leader sequence (SL) gene, complete sequence	65.8	65.8	100%	2e-08	97%	KJ474905.1
Leishmania mexicana strain M379 hexose transporter (D2) mRNA, complete cds	65.8	65.8	89%	2e-08	100%	EU449769.1
Trypanosomatidae sp. 97SI trans-spliced leader sequence gene, complete sequence	65.8	65.8	100%	2e-08	97%	DQ864301.1
Leptomonas pyrrhocoris trans-spliced leader sequence SL gene, complete sequence	65.8	65.8	100%	2e-08	97%	DQ140169.1
Leishmania major hexokinase mRNA, partial cds	65.8	65.8	89%	2e-08	100%	AY702029.1
p17=kinetoplast DNA-associated protein [Crithidia fasciculata, mRNA Kinetoplast Partial, 233 nt]	65.8	65.8	89%	2e-08	100%	S56496.1
p16=kinetoplast DNA-associated protein [Crithidia fasciculata, mRNA Kinetoplast Partial, 250 nt]	65.8	65.8	89%	2e-08	100%	S56498.1
Leishmania donovani DNA topoisomerase II (TOP2) gene, complete cds	65.8	65.8	89%	2e-08	100%	AF150876.2
Leishmania donovani cyclophilin (CYP) gene, complete cds	65.8	65.8	89%	2e-08	100%	AF158368.1
Leishmania mexicana gene for carbamyl phosphate synthase, complete cds	65.8	65.8	89%	2e-08	100%	AB005062.1
L.mexicana leading sequence of a transcript created by recombination	65.8	65.8	89%	2e-08	100%	Y07805.1
Crithidia fasciculata calcium binding protein mRNA, partial cds	65.8	65.8	89%	2e-08	100%	U21305.1
Leptomonas seymouri alpha-tubulin mRNA 5' end	65.8	65.8	89%	2e-08	100%	X14005.1
Leptomonas barvae isolate 17EC-A trans-spliced leader sequence gene, complete sequence	63.9	63.9	94%	8e-08	97%	DQ864310.1
Leishmania donovani promastigote surface antigen (PSAa) mRNA, partial cds	63.9	63.9	87%	8e-08	100%	DQ086114.1
Leishmania enriettii alpha-tubulin mRNA, partial cds	63.9	63.9	87%	8e-08	100%	K03548.1

Description	Max score	Total score	Query cover	E value	Ident	Accession
Leishmania braziliensis guayanensis 5' end of medRNA	63.9	63.9	87%	8e-08	100%	X15946.1
Leishmania peruviana genome assembly Leishmania peruviana PAB-4377_V1, chromosome : 16	62.1	178	100%	3e-07	95%	LN609244.1
Leishmania peruviana genome assembly Leishmania peruviana PAB-4377_V1, chromosome : 8	62.1	116	100%	3e-07	95%	LN609243.1
Leishmania braziliensis MHOM/BR/75/M2904 complete genome, chromosome 16	62.1	233	100%	3e-07	95%	FR798990.1
Leishmania braziliensis MHOM/BR/75/M2904 complete genome, chromosome 8	62.1	116	100%	3e-07	95%	FR798982.1
Leishmania braziliensis MHOM/BR/75/M2904 complete genome, chromosome 2	62.1	62.1	100%	3e-07	95%	FR798976.1
Leishmania amazonensis isolate MHOM/BR/1973/M2269 phosphatidylserine synthase (PSSII) mRNA, complete cds	62.1	62.1	84%	3e-07	100%	EU706336.1
Leishmania braziliensis strain MHOM/BR/75/M2904 alpha tubulin mRNA, 5' UTR	62.1	62.1	84%	3e-07	100%	FJ750454.1
Crithidia insperata isolate 119YS trans-spliced leader sequence SL (trans-spliced leader sequence SL) gene, complete sequence	62.1	62.1	84%	3e-07	100%	EU088273.1
Leishmania amazonensis clone La758.1 external transcribed spacer, complete sequence; and small subunit ribosomal RNA, partial sequence	62.1	62.1	84%	3e-07	100%	DQ907229.1
Crithidia fasciculata clone Cf67.12 external transcribed spacer, complete sequence; and small subunit ribosomal RNA, partial sequence	62.1	62.1	84%	3e-07	100%	DQ907228.1
Crithidia fasciculata clone Cf67.5 external transcribed spacer, complete sequence; and small subunit ribosomal RNA, partial sequence	62.1	62.1	84%	3e-07	100%	DQ907227.1
Leishmania donovani chagasi glucose-6-phosphate dehydrogenase mRNA, partial cds	62.1	62.1	84%	3e-07	100%	DQ212794.1
Leishmania infantum glucose-6-phosphate dehydrogenase mRNA, partial cds	62.1	62.1	84%	3e-07	100%	DQ212793.1
Trypanosomatidae sp. 52CR-B trans-spliced leader sequence, complete sequence	62.1	62.1	100%	3e-07	95%	DQ860231.1
Leptomonas pyrrhocoris isolate 28EC-B trans-spliced leader sequence gene, complete sequence	62.1	62.1	100%	3e-07	95%	DQ860219.1
Crithidia fasciculata mRNA for NIMA-related kinase (nek gene)	62.1	62.1	84%	3e-07	100%	AJ494838.1
Leishmania peruviana strain MHOM/PE/1984/LC39 glucose-6-phosphate dehydrogenase (G6PD) mRNA, partial cds	62.1	62.1	84%	3e-07	100%	AY099306.1
Leishmania panamensis strain MHOM/PA/1971/LS94 glucose-6-phosphate dehydrogenase (G6PD) mRNA, partial cds	62.1	62.1	84%	3e-07	100%	AY099305.1
Leishmania lainsoni strain MHOM/BR/1981/M6426 glucose-6-phosphate dehydrogenase (G6PD) mRNA, partial cds	62.1	62.1	84%	3e-07	100%	AY099304.1

Description	Max score	Total score	Query cover	E value	Ident	Accession
Leishmania shawi strain MCEB/BR/1984/M8408 glucose-6-phosphate dehydrogenase (G6PD) mRNA, partial cds	62.1	62.1	84%	3e-07	100%	AY099303.1
Leishmania naiffi strain MDAS/BR/1979/M5533 glucose-6-phosphate dehydrogenase (G6PD) mRNA, partial cds	62.1	62.1	84%	3e-07	100%	AY099302.1
Leishmania braziliensis strain MHOM/BR/1975/M2903 glucose-6-phosphate dehydrogenase (G6PD) mRNA, partial cds	62.1	62.1	84%	3e-07	100%	AY099301.1
Leishmania guyanensis strain MHOM/BR/1975/M4147 glucose-6-phosphate dehydrogenase (G6PD) mRNA, complete cds	62.1	62.1	84%	3e-07	100%	AY099300.1
Leishmania mexicana strain MHOM/BZ/1982/BEL21 glucose-6-phosphate dehydrogenase (G6PD) mRNA, complete cds	62.1	62.1	84%	3e-07	100%	AY099299.1
Leishmania mexicana amazonensis strain MHOM/BR/1973/M2269 glucose-6-phosphate dehydrogenase (G6PD) mRNA, complete cds	62.1	62.1	84%	3e-07	100%	AY099298.1
Crithidia fasciculata 27 kDa guide RNA-binding protein mRNA, complete cds; mitochondrial gene for mitochondrial product	62.1	62.1	84%	3e-07	100%	AF157559.1
Leishmania infantum 22 kDa potentially aggravating protein papLe22 mRNA, complete cds	62.1	62.1	100%	3e-07	95%	AF123892.1
Leishmania amazonensis clone AR26 demethoxyubiquinone hydroxylases (DMQH)-like mRNA, partial sequence	60.2	60.2	82%	1e-06	100%	EU429375.1
p18=kinetoplast DNA-associated protein [Crithidia fasciculata, mRNA Kinetoplast Partial, 192 nt]	60.2	60.2	82%	1e-06	100%	S56494.1
Leishmania tarentolae DNA for mini-exon, 485 bp	60.2	60.2	82%	1e-06	100%	X97619.1
Leptomonas seymouri mini-exon with insertion element LINS1	58.4	114	100%	4e-06	100%	X07488.1
Leishmania sp. LECU1 trans-spliced leader-like min-exon, partial sequence	56.5	56.5	76%	1e-05	100%	GQ293227.1
Leishmania panamensis strain MHOM/PA/71/LS94 mini-exon repeat	56.5	56.5	76%	1e-05	100%	AY155509.1
Leishmania mexicana strain MNYC/BZ/62/M379 mini-exon repeat	56.5	56.5	76%	1e-05	100%	AY155508.1
Leishmania mexicana venezuelensis strain MHOM/VE/74/PM-H3 mini-exon repeat	56.5	56.5	76%	1e-05	100%	AY155507.1
Leishmania peruviana strain MHOM/PE/84/LC39 mini-exon repeat	56.5	56.5	76%	1e-05	100%	AY155506.1
Leishmania killicki strain MHOM/TN/80/LEM163 mini-exon repeat	56.5	56.5	76%	1e-05	100%	AY155504.1
Leishmania infantum strain MHOM/FR/78/LEM75 mini-exon repeat	56.5	56.5	76%	1e-05	100%	AY155503.1
Leishmania colombiensis strain IGOM/PA/85/E582.34 mini-exon repeat	56.5	56.5	76%	1e-05	100%	AY155502.1
Leishmania aethiopica strain MHOM/ET/72/L100 mini-exon repeat	56.5	56.5	76%	1e-05	100%	AY155501.1
Leishmania donovani DNA, mini-exon	56.5	56.5	76%	1e-05	100%	AB162853.1
Leishmania panamensis DNA, trans-spliced leader RNA sequence, strain:G-05	56.5	56.5	76%	1e-05	100%	AB093592.1
Endotrypanum schaudinni DNA, trans-spliced leader RNA sequence	56.5	56.5	76%	1e-05	100%	AB092603.1

Description	Max score	Total score	Query cover	E value	Ident	Accession
Endotrypanum monterogeii DNA, trans-spliced leader RNA sequence	56.5	56.5	76%	1e-05	100%	AB092602.1
Endotrypanum sp. M6843 LV-59 DNA, trans-spliced leader RNA sequence	56.5	56.5	76%	1e-05	100%	AB092601.1
Endotrypanum sp. M6842 LV-58 DNA, trans-spliced leader RNA sequence	56.5	56.5	76%	1e-05	100%	AB092600.1
Endotrypanum sp. OC-4 DNA, trans-spliced leader RNA sequence	56.5	56.5	76%	1e-05	100%	AB092599.1
Endotrypanum sp. OC-3 DNA, trans-spliced leader RNA sequence	56.5	56.5	76%	1e-05	100%	AB092598.1
Endotrypanum sp. OC-2 DNA, trans-spliced leader RNA sequence	56.5	56.5	76%	1e-05	100%	AB092597.1
Leishmania equatorensis DNA, trans-spliced leader RNA sequence, strain:Lsp-2	56.5	56.5	76%	1e-05	100%	AB092596.1
Leishmania equatorensis DNA, trans-spliced leader RNA sequence, strain:Lsp-1	56.5	56.5	76%	1e-05	100%	AB092595.1
Crithidia luciliae thermophila mini-exon gene repeat	56.5	56.5	76%	1e-05	100%	U96171.1
Crithidia acanthocephali mini-exon gene repeat	56.5	56.5	76%	1e-05	100%	U96167.1
L.lainsoni mini-exon gene for medRNA	56.5	56.5	76%	1e-05	100%	X77913.1
Phytomonas serpens (mini-exon derived RNA) mini-exon region	56.5	56.5	76%	1e-05	100%	L05396.1
Leishmania amazonensis mini-exons	56.5	56.5	76%	1e-05	100%	L05000.1
Leishmania amazonensis (mini-exon derived RNA) mini-exon region	56.5	56.5	76%	1e-05	100%	L05395.1
Endotrypanum schaudinni (mini-exon derived RNA) mini-exon region	56.5	56.5	76%	1e-05	100%	L05398.1
Endotrypanum schaudinni (mini-exon derived RNA) mini-exon region	56.5	56.5	76%	1e-05	100%	L05397.1
L.tropica (MHOM/Sudan/58/OD) mini-exon	56.5	56.5	76%	1e-05	100%	X69451.1
L.shawi (MCEB/Brazil/84/M8408) mini-exon	56.5	56.5	76%	1e-05	100%	X69455.1
L.panamensis (MHOM/Panama/71/LS94) mini-exon	56.5	56.5	76%	1e-05	100%	X69450.1
L.major (MHOM/Israel/83/LT252) mini-exon	56.5	56.5	76%	1e-05	100%	X69449.1
L.mexicana (MHOM/Belize/82/BEL21) mini-exon	56.5	56.5	76%	1e-05	100%	X69447.1
L.mexicana (MNYC/Belize/62/M379) mini-exon	56.5	56.5	76%	1e-05	100%	X69444.1
L.infantum (MHOM/Tunisia/80/IPTI) mini-exon	56.5	56.5	76%	1e-05	100%	X69445.1
L.guyanensis (MHOM/Brazil/75/M4147) mini-exon	56.5	56.5	76%	1e-05	100%	X69452.1
Leishmania enviettii spliced leader gene	56.5	56.5	76%	1e-05	100%	X04378.1
L.donovani (MHOM/Kenya730/MRC-L74) mini-exon (small gene)	56.5	56.5	76%	1e-05	100%	X69456.1
L.donovani (MHOM/India/80/DD8) mini-exon (small gene)	56.5	56.5	76%	1e-05	100%	X69453.1
L.donovani (MHOM/India/80/DD8) mini-exon (larger gene)	56.5	56.5	76%	1e-05	100%	X69443.1
L.chagasi (MHOM/Brazil/82/BA3) mini-exon	56.5	56.5	76%	1e-05	100%	X69446.1
L.brasiliensis (LTB 300) mini-exon	56.5	56.5	76%	1e-05	100%	X69442.1
L.brasiliensis (MHOM/Brazil/75/M2903) mini-exon	56.5	56.5	76%	1e-05	100%	X69441.1

Description	Max score	Total score	Query cover	E value	Ident	Accession
L.aethiopica MINI-exon; mini-exon donor RNA	56.5	56.5	76%	1e-05	100%	X68788.1
Leishmania panamensis strain MHOM/PA/94/PSC-1 chromosome 2 sequence	54.7	54.7	74%	5e-05	100%	CP009371.1
Leishmania sp. AM-2004 mini-exon gene tandem array	54.7	54.7	74%	5e-05	100%	AY495831.1
Leishmania panamensis histone H1 mRNA, complete cds	54.7	54.7	74%	5e-05	100%	AY356348.1
Leishmania panamensis histone H1 mRNA, complete cds	54.7	54.7	74%	5e-05	100%	AY356347.1
Leishmania amazonensis cP36 mRNA, complete cds	54.7	54.7	74%	5e-05	100%	U38835.1
Leishmania chagasi surface membrane protein GP46 mRNA, complete cds	52.8	52.8	71%	2e-04	100%	AF006588.1

Alignments

Uncultured trypanosome isolate 435AR-B-env trans-spliced leader sequence (SL) gene, complete sequence
Sequence ID: **KR056279.1** Length: 269 Number of Matches: 1
Range 1: 1 to 39

Score	Expect	Identities	Gaps	Strand	Frame
73.1 bits(39)	1e-10()	39/39(100%)	0/39(0%)	Plus/Plus	

Features:

```
Query  1    AACTAACGCTATATAAGTATCAGTTTCTGTACTTTATTG  39
            |||||||||||||||||||||||||||||||||||||||
Sbjct  1    AACTAACGCTATATAAGTATCAGTTTCTGTACTTTATTG  39
```

Uncultured trypanosome isolate 434AR-B-env trans-spliced leader sequence (SL) gene, complete sequence
Sequence ID: **KR056277.1** Length: 269 Number of Matches: 1
Range 1: 1 to 39

Score	Expect	Identities	Gaps	Strand	Frame
73.1 bits(39)	1e-10()	39/39(100%)	0/39(0%)	Plus/Plus	

Features:

```
Query  1    AACTAACGCTATATAAGTATCAGTTTCTGTACTTTATTG  39
            |||||||||||||||||||||||||||||||||||||||
Sbjct  1    AACTAACGCTATATAAGTATCAGTTTCTGTACTTTATTG  39
```

Uncultured trypanosome isolate 425LB-env trans-spliced leader sequence (SL) gene, complete sequence
Sequence ID: **KR056271.1** Length: 269 Number of Matches: 1
Range 1: 1 to 39

Score	Expect	Identities	Gaps	Strand	Frame
73.1 bits(39)	1e-10()	39/39(100%)	0/39(0%)	Plus/Plus	

Features:

```
Query  1    AACTAACGCTATATAAGTATCAGTTTCTGTACTTTATTG  39
            |||||||||||||||||||||||||||||||||||||||
Sbjct  1    AACTAACGCTATATAAGTATCAGTTTCTGTACTTTATTG  39
```

Uncultured trypanosome isolate 405LB-env trans-spliced leader sequence (SL) gene, complete sequence
Sequence ID: **KR056263.1** Length: 513 Number of Matches: 1
Range 1: 1 to 39

Score	Expect	Identities	Gaps	Strand	Frame
73.1 bits(39)	1e-10()	39/39(100%)	0/39(0%)	Plus/Plus	

Features:

```
Query  1    AACTAACGCTATATAAGTATCAGTTTCTGTACTTTATTG  39
            |||||||||||||||||||||||||||||||||||||||
Sbjct  1    AACTAACGCTATATAAGTATCAGTTTCTGTACTTTATTG  39
```

Uncultured trypanosome isolate 404LB-env trans-spliced leader sequence (SL) gene, complete sequence
Sequence ID: **KR056262.1** Length: 513 Number of Matches: 1
Range 1: 1 to 39

Score	Expect	Identities	Gaps	Strand	Frame
73.1 bits(39)	1e-10()	39/39(100%)	0/39(0%)	Plus/Plus	

Features:

```
Query  1    AACTAACGCTATATAAGTATCAGTTTCTGTACTTTATTG  39
            |||||||||||||||||||||||||||||||||||||||
Sbjct  1    AACTAACGCTATATAAGTATCAGTTTCTGTACTTTATTG  39
```

BLAST is a registered trademark of the National Library of Medicine

Supplementary Material S3

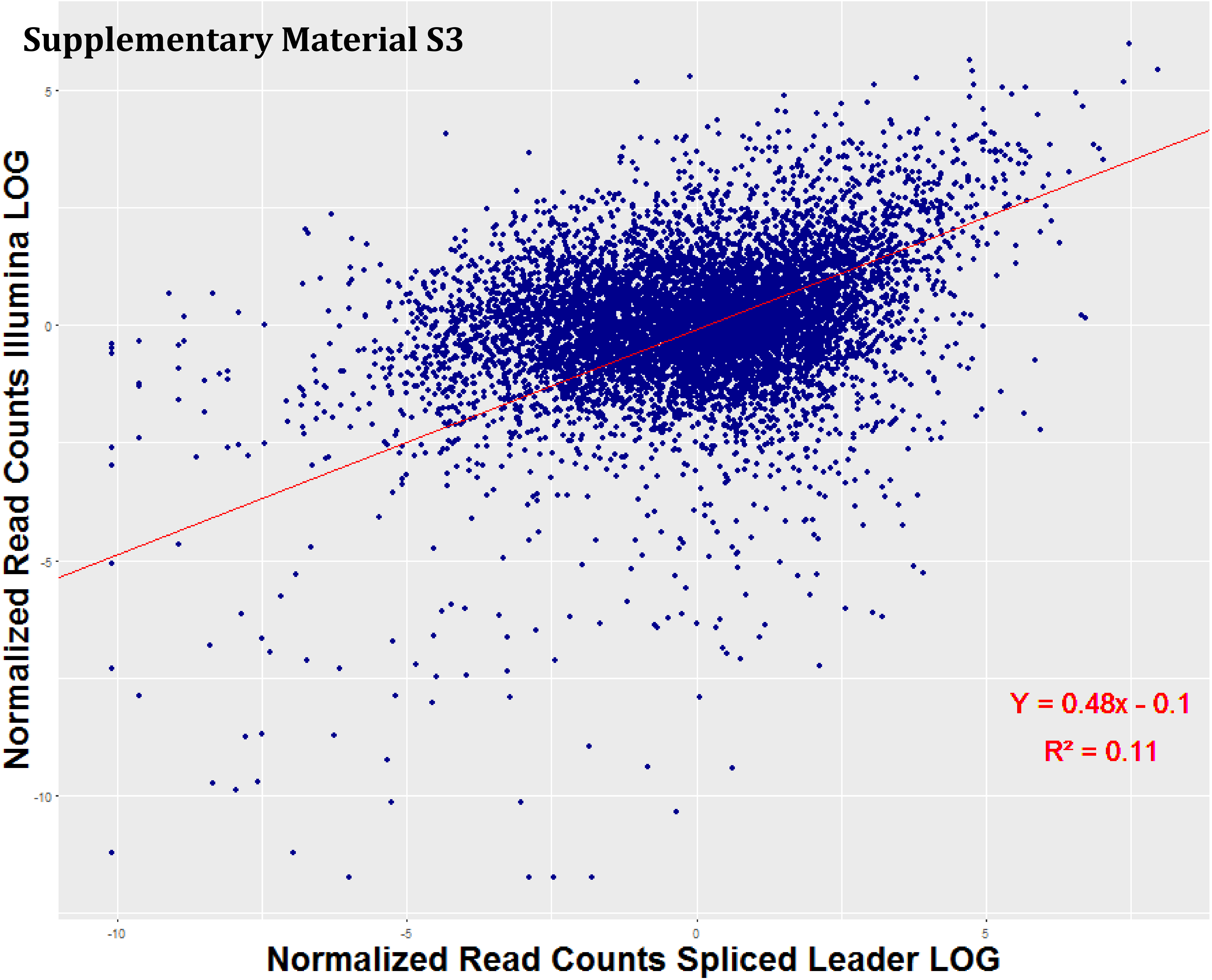

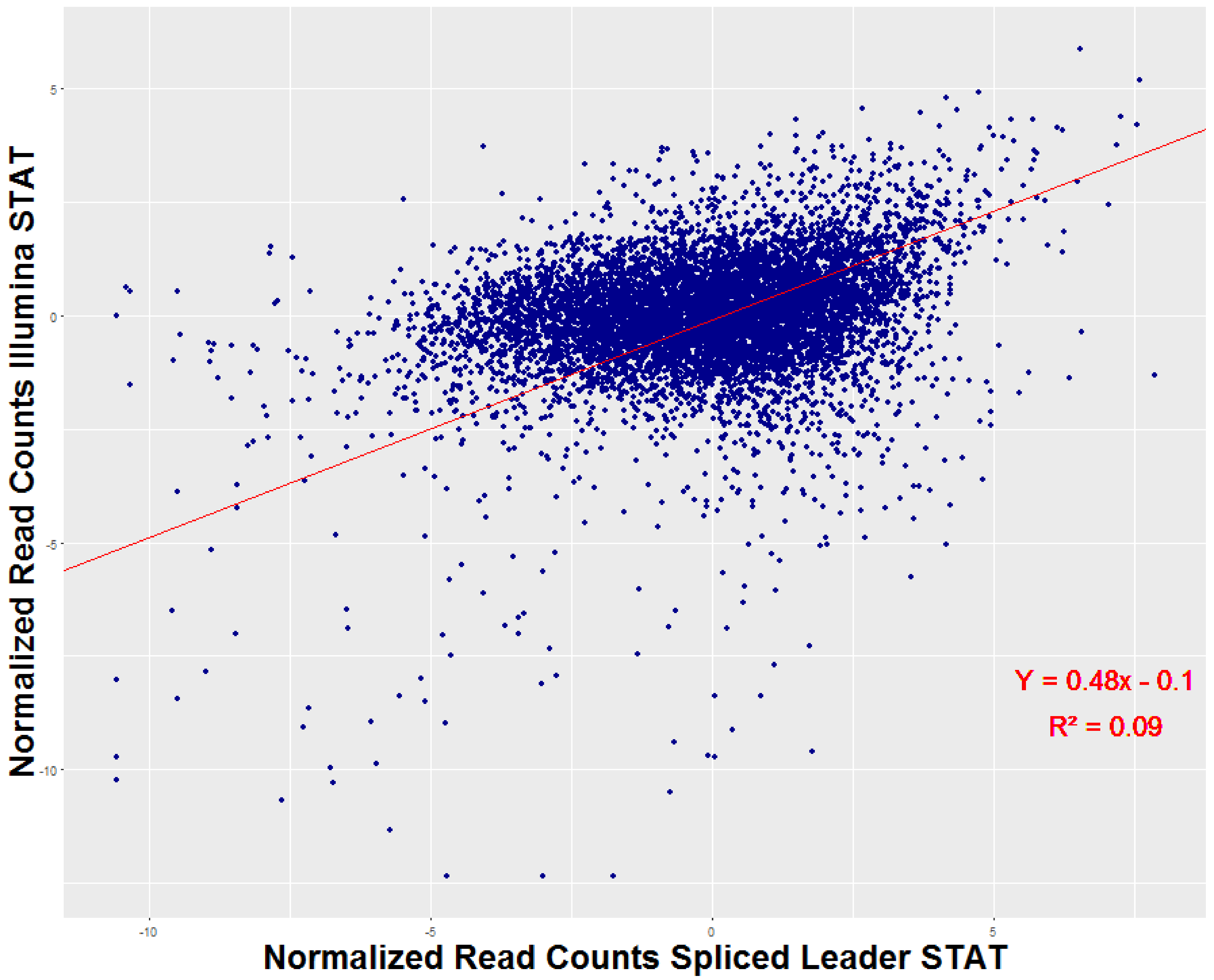

Normalized Read Counts Illumina STAT
5
0
-5
-10
-10
-5
0
5
Normalized Read Counts Spliced Leader STAT
Y = 0.48x - 0.1
R² = 0.09

Supplementary Material S4

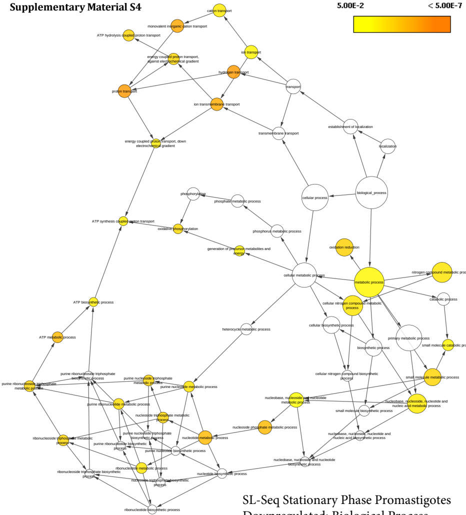

SL-Seq Stationary Phase Promastigotes
Downregulated: Biological Process

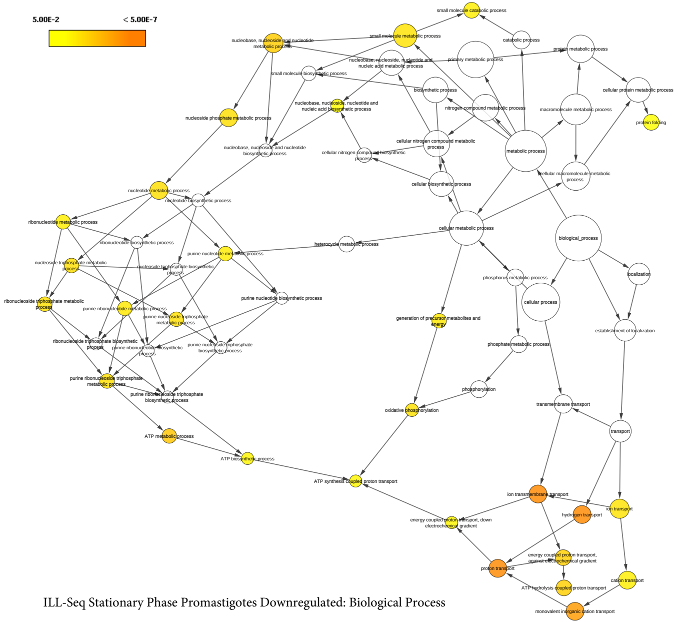

ILL-Seq Stationary Phase Promastigotes Downregulated: Biological Process

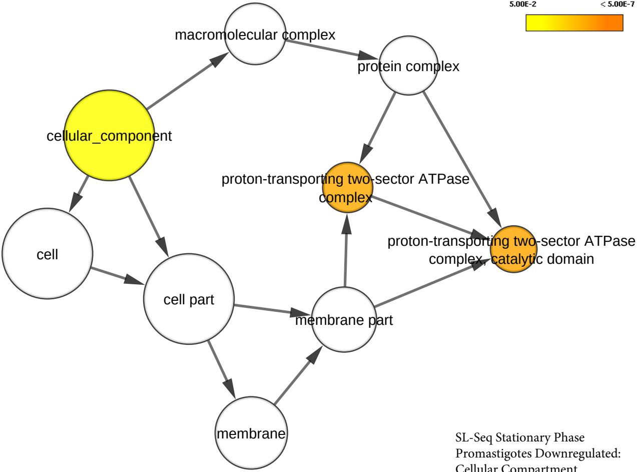

SL-Seq Stationary Phase Promastigotes Downregulated: Cellular Compartment

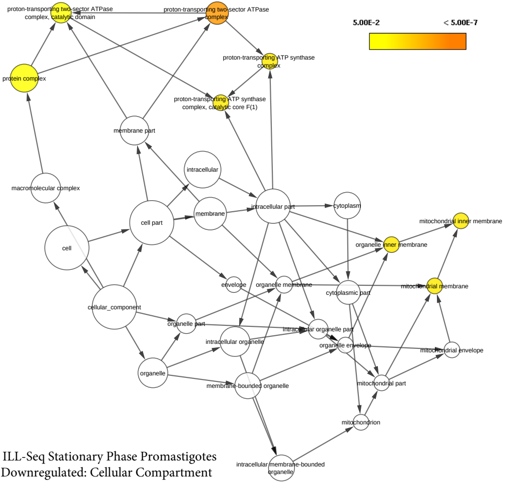

ILL-Seq Stationary Phase Promastigotes
Downregulated: Cellular Compartment

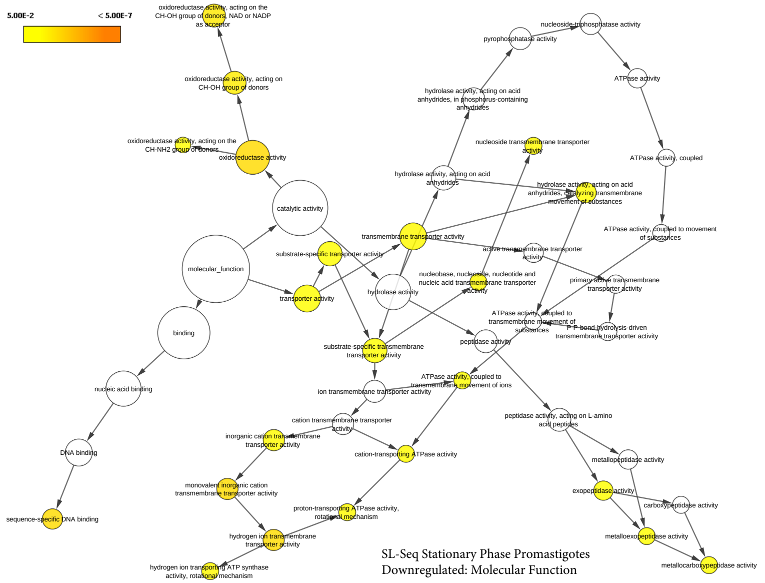

SL-Seq Stationary Phase Promastigotes Downregulated: Molecular Function

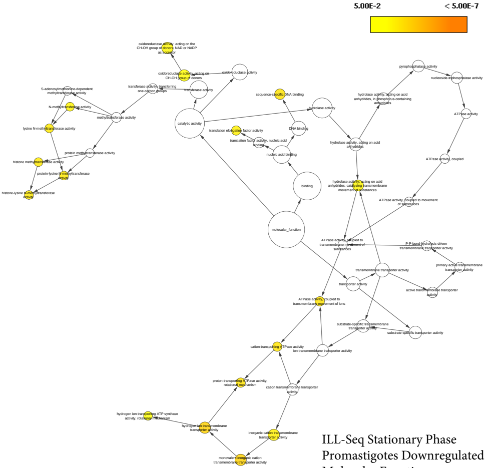

ILL-Seq Stationary Phase Promastigotes Downregulated: Molecular Function

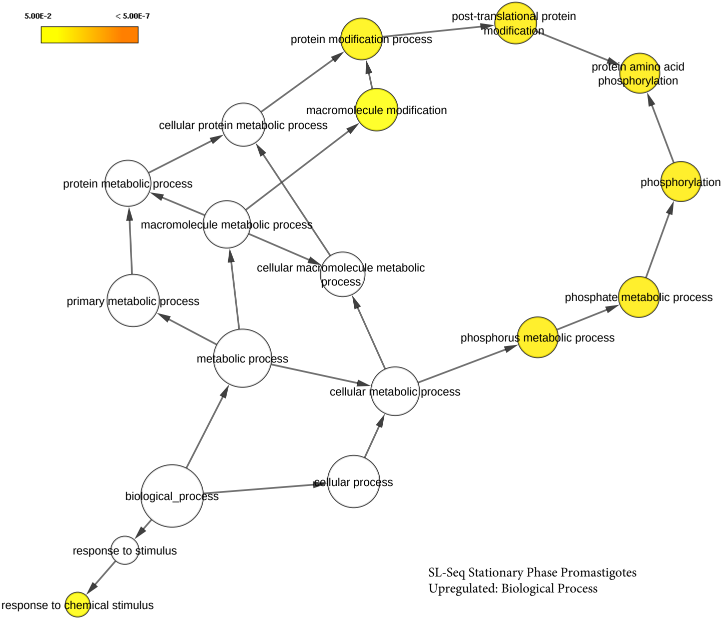

SL-Seq Stationary Phase Promastigotes
Upregulated: Biological Process

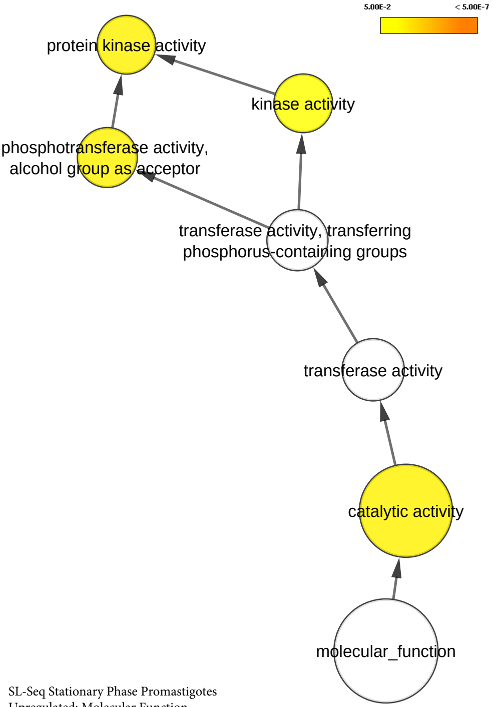

SL-Seq Stationary Phase Promastigotes
Upregulated: Molecular Function

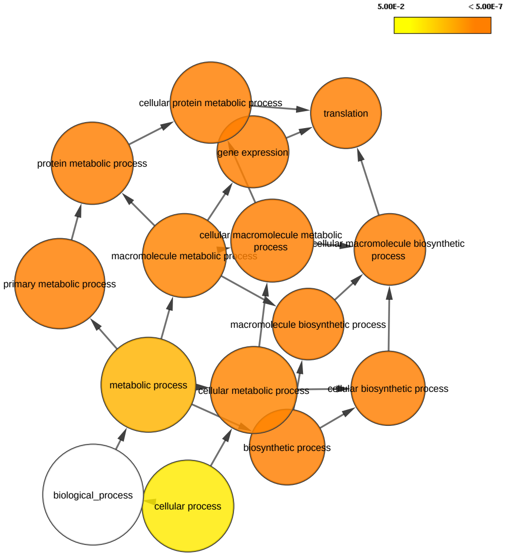

SL-Seq Amastigotes Downregulated: Biological Process

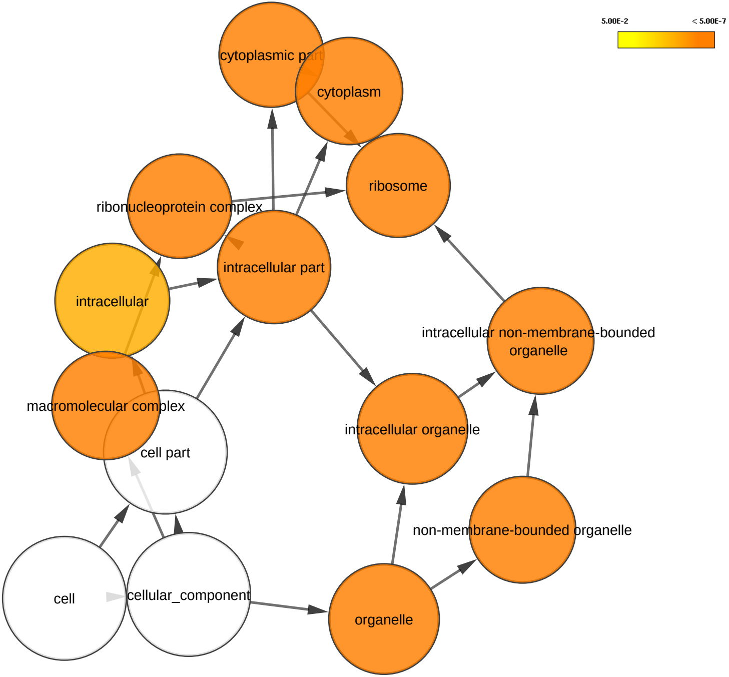

SL-Seq Amastigotes Downregulated: Cellular Compartement

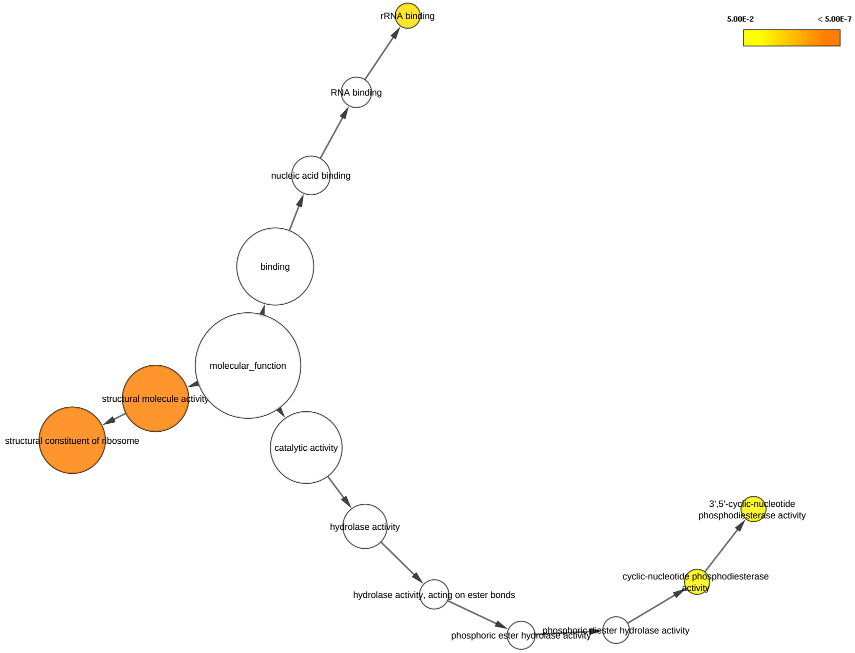

SL-Seq Amastigotes Downregulated: Molecular Function

Supplementary Material S5

Downregulated STAT	Shared SL-Seq / ILL-Seq	SL-Seq / ILL-Seq Unique
ATP-Synthase	beta subunit (LdBPK_250017800 & LdBPK_250017900), delta subunit (LdBPK_300043400), subunit c (LdBPK_210013200), epsilon subunit (LdBPK_360011900)	(OSCP) subunit (LdBPK_360080100) / gamma subunit (LdBPK_210027100)
Transmembrane proton transport	V-ATPase subunit c (LdBPK_180010500), subunit b (LdBPK_280031400), subunit e (LdBPK_360039400), vacuolar-type proton translocating pyrophosphatase 1 (LdBPK_310019800)	
Oxidoreductase activity with NAD or NADP as electron donor	isocitrate hydrogenase (LdBPK_100008300), LdBPK_130022000, LdBPK_230009000, malate hydrogenase (LdBPK_240012900), LdBPK_300006300, LdBPK_300039000, LdBPK_330035000, LdBPK_340021100, LdBPK_360048300	LdBPK_300039000, LdBPK_330035000 / LdBPK_330032400
Sequence-specific DNA binding	histone H4 genes (LdBPK_310042200, LdBPK_360005100, LdBPK_150005000), histone H3 genes (LdBPK_100015500 & LdBPK_100017100)	histon H4 (LdBPK_060005000) / Core histone-like transcription factor (CBF/NF-Y) (LdBPK_160010700)
Transcription elongation	Translation elongation factor 1 beta (LdBPK_360020600), elongation factor 1 gamma (LdBPK_090015600)	eukaryotic initiation factor 5a (LdBPK_250013000), elongationfactor1-beta (LdBPK_340014000), translation,

Protein folding	HSP90 (LdBPK_290012400), HSP10 (LdBPK_260011300), Cyclophilin 40 (LdBPK_350053500), Cyclophilin-type peptidyl-prolyl cis-trans isomerase (LdBPK_250014800), prefoldin subunit 2 (LdBPK_050017200), calreticulin (LdBPK_310035300)	co-chaperone GrpE (LdBPK_300012700), Peptidyl-prolyl cis-trans isomerase (LdBPK_360007600)
Upregulated STAT		
Protein phosphorylation	Protein kinases (LdBPK_320013200, LdBPK_360030200, LdBPK_270023600, LdBPK_080018000, LdBPK_200014400, LdBPK_040017600, LdBPK_080014800, LdBPK_320007600)	Protein kinases (LdBPK_170011000, LdBPK_210025800, LdBPK_360055100, LdBPK_350057600, LdBPK_260026400, LdBPK_300008900, LdBPK_320023900, , LdBPK_170015100)
